# Suppressing ACQ of molecular photosensitizers by distorting the conjugated-plane for enhanced tumor photodynamic therapy†

**DOI:** 10.1039/d3sc05041f

**Published:** 2023-12-12

**Authors:** Han Sun, Lukun Li, Ruihua Guo, Zhe Wang, Yanhui Guo, Zhiliang Li, Fengling Song

**Affiliations:** a Institute of Molecular Science and Engineering, Institute of Frontier and Interdisciplinary Science, Shandong University Qingdao Shandong 266237 China zhiliang.li@sdu.edu.cn songfl@sdu.edu.cn; b Department of Materials Science and Engineering, Hainan University Haikou Hainan 570228 China wangzhe@hainanu.edu.cn

## Abstract

Non-AIE-type molecular photosensitizers (PSs) suffer from the aggregation-caused-quenching (ACQ) effect in an aqueous medium due to the strong hydrophobic and π–π interactions of their conjugated planes, which significantly hinders the enhancement of tumor photodynamic therapy (PDT). So far, some ionic PSs have been reported with good water-solubility, though the ACQ effect can still be induced in a biological environment rich in ions, leading to unsatisfactory *in vivo* delivery and fluorescence imaging performance. Hence, designing molecular PSs with outstanding anti-ACQ properties in water is highly desirable, but it remains a tough challenge for non-AIE-type fluorophores. Herein, we demonstrated a strategy for the design of porphyrin-type molecular PSs with remarkable solubility and anti-ACQ properties in an aqueous medium, which was assisted by quantum chemical simulations. It was found that cationic branched side chains can induce serious plane distortion in diphenyl porphyrin (DPP), which was not observed for tetraphenyl porphyrin (TPP) with the same side chains. Moreover, the hydrophilicity of the chain spacer is also crucial to the plane distortion for attaining the desired anti-ACQ properties. Compared to ACQ porphyrin, anti-ACQ porphyrin displayed type-I ROS generation in hypoxia and much higher tumor accumulation efficacy by blood circulation, leading to highly efficient *in vivo* PDT for hypoxic tumors. This study demonstrates the power of sidechain chemistry in tuning the configuration and aggregation behaviors of porphyrins in water, offering a new path to boost the performance of PSs to fulfill the increasing clinical demands on cancer theranostics.

## Abstract 2

This work demonstrates a novel strategy to address the notorious ACQ of non-AIE type fluorophores in an aqueous medium through deep insights into the structural effects leading to anti-ACQ properties, which could benefit the potential applications.

## Graphical abstract

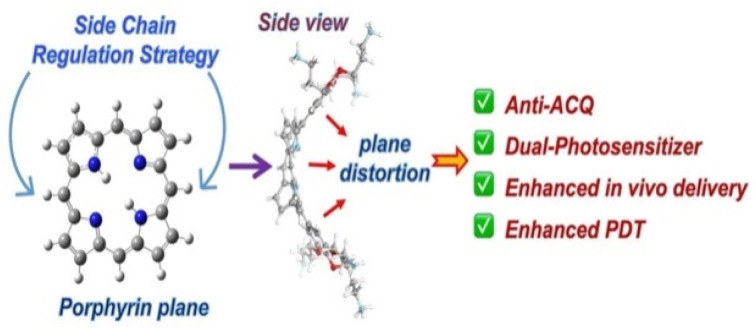

## Introduction

Photodynamic therapy (PDT) has attracted great attention in curing cancers due to its minimal invasiveness and side-effects, spatiotemporal selectivity and high therapeutic efficacy compared to conventional cancer treatments.^1–6^ In PDT, photoexcited photosensitizers (PSs) convert triplet oxygen and other molecules into cytotoxic reactive oxygen species (ROS) *via* electron transfer (type-I) or energy transfer (type-II), which subsequently induces apoptosis or necrosis of tumor cells.^7–11^ However, the high hydrophobicity and aggregation tendency of molecular PSs under physiological conditions result in lowered ROS generation and poor *in vivo* delivery efficacy.^6,7,12,13^ Significant efforts have been dedicated to alleviating these limitations of molecular PSs for PDT by involving additional bioactive components and carriers to fabricate nanomedicines, which can complicate the whole process and may cause undesired side effects.^14–16^

Although a large variety of AIE-type nano-PSs have been reported to address the issues caused by aggregation-caused-quenching (ACQ) of fluorophores, this is not applicable to the other traditional fluorescent molecules.^17–21^ For example, for one of the most valuable PSs, porphyrins, which are extensively used for cancer treatment in clinics owing to their excellent optical properties, photostability and biocompatibility, the aforementioned limitations have not been successfully solved.^6^ Porphyrins severely suffer from notorious superhydrophobicity, and extremely tend to aggregate in an aqueous medium, causing serious reduction in fluorescence emission and poor *in vivo* delivery efficacy.^6,22,23^ To date, some PSs decorated with cationic side chains have been reported for PDT of tumors and drug-resistant bacteria.^24,25^ Due to the excellent hydrophilicity of positive charges, cationic PSs usually exhibit enhanced water-solubility and are more easily taken up by cancer cells than normal cells due to the more electronegative membrane of cancer cells. At the same time, cationic PSs can also target the suborganelles of cancer cells, which can promote a series of inflammatory and immune responses by the ROS produced during PDT, destroy the tumor microvasculature, and ultimately induce tumor cell death.^26–28^

However, cationic PSs can still be induced to aggregate by the physiological environment, biological polyanions (*e.g.*, adenosine triphosphates (ATP) and pyrophosphate (PPi)), and high ionic strength, resulting in serious fluorescence quenching which greatly limits the potential *in vivo* applications.^7,29,30^ Therefore, it is very promising to develop cationic molecular PSs with excellent anti-ACQ properties in a physiological environment. Recently, it was shown that porphyrins can be restricted in rigid inorganic, metal–organic or polymeric frameworks to prevent aggregation; however, this leads to very poor water-solubility.^31–33^ Moreover, a strategy for designing molecular porphyrins with excellent anti-ACQ properties in a physiological environment has not been reported. It has been shown that the 5, 15-diphenyl porphyrin exhibits conjugated plane distortion, and the steric effect from substituents on *β*-pyrrole could also distort the porphyrin plane.^34,35^ We speculate that introducing bulky cationic side chains at the 5- and 15- positions may cause more serious distortion of the porphyrin plane which weakens the intermolecular π–π interactions, thereby avoiding the ACQ effect in water even with polyanions or high ionic strength. Therefore, cationic porphyrins with a distorted plane and bulky side chains would possess anti-ACQ properties in a physiological environment which benefits *in vivo* tumor PDT performance.

**Fig. 1:**
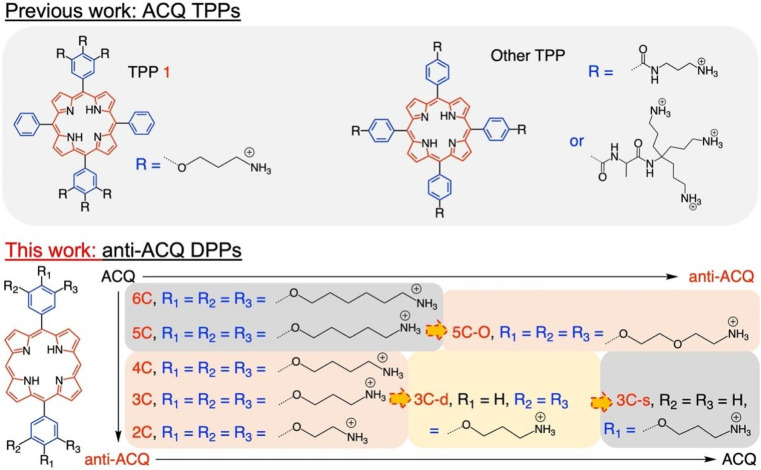
Chemical structures of some previously reported ACQ cationic TPPs and the DPP-type cationic porphyrins studied in this work. (Naming rules for DPP: the numbers denote the length of each chain spacer, the capital letters denote the atoms in the chain spacer, and the lowercase letters denote the number of side chains on each phenyl ring. s: single and d: double).

**Scheme 1:**
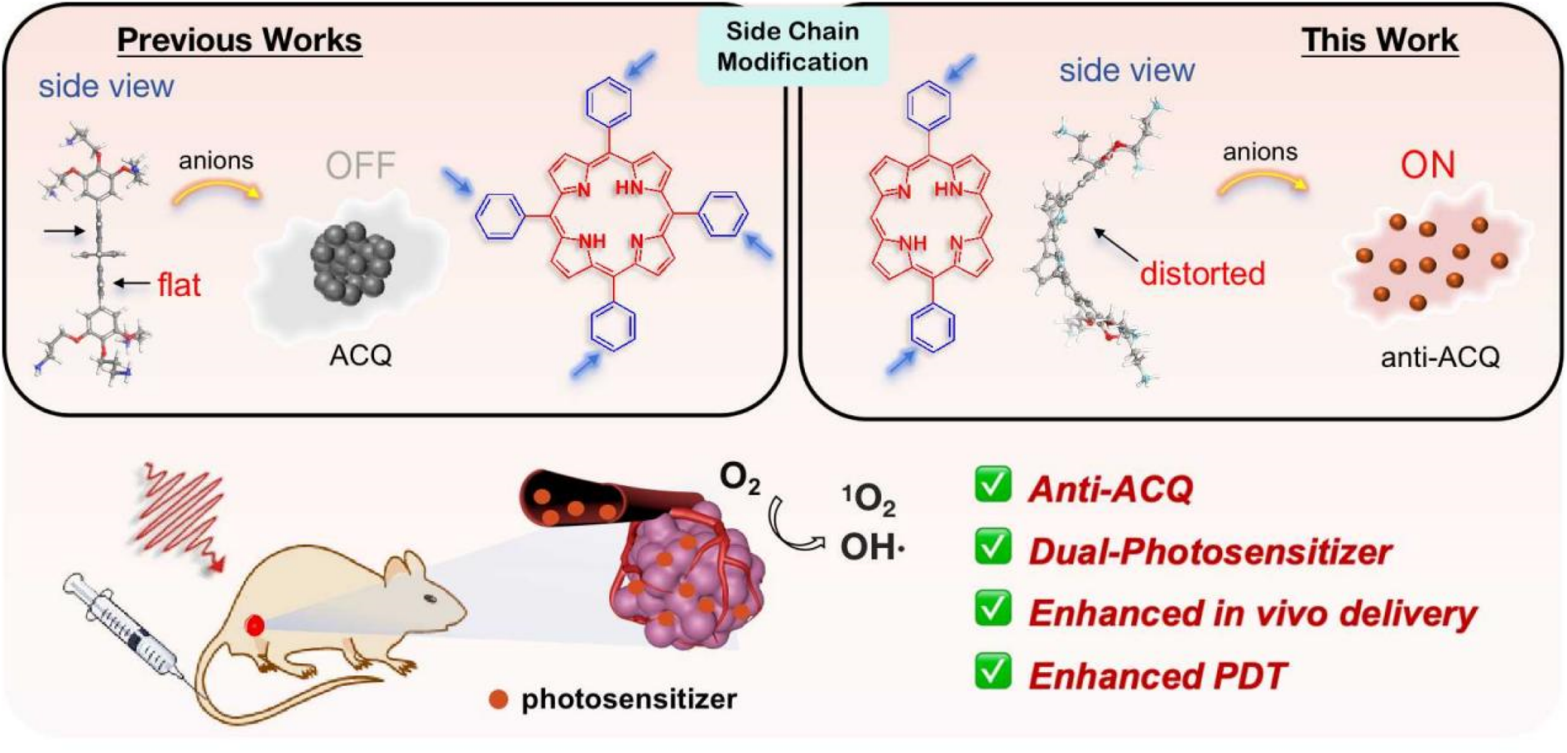
Schematic illustration of designing anti-ACQ porphyrin in an aqueous medium for enhanced PDT of hypoxic tumors.

Herein, we demonstrate a strategy for designing molecular PSs with excellent solubility and anti-ACQ properties in water. A series of water-soluble diphenylporphyrin (DPP)-based PSs (Fig. 1) substituted with cationic side chains varying in number, length and hydrophilicity were synthesized and investigated. DPP with branched side chains exhibited distorted conjugated planes and the extent of plane distortion is associated with the hydrophilicity of the chain spacer. A significantly more distorted plane was observed for DPP with a shorter carbon chain (C_3_*vs.* C_6_) in quantum chemical calculations. The plane distortion endows DPP 3C with unique anti-ACQ properties with polyanions and high ionic strength, allowing its high ROS generation efficacy in both type-I (OH˙) and type-II (^1^O_2_) pathways, and strong photocytotoxicity to cancer cells in both normoxia and hypoxia. Moreover, much more efficient *in vivo* delivery of 3C to the tumor site is achieved through whole-body blood circulation compared to ACQ porphyrin TPP 1, leading to great PDT performance. The unique properties of the anti-ACQ cationic porphyrin allow one-molecule-based cancer therapy with high precision and efficacy, which is highly beneficial to the diagnosis and treatment of cancers (Scheme 1).

## Results and discussion

### Synthesis

The chemical structures of the novel porphyrins presented in this work are based on 5,15-diphenylporphyrin (DPP) with varying number, length or composition of cationic side chains linked to each phenyl ring *via* ether bonds (Fig. 1). The detailed synthetic protocols of these porphyrins are described in the ESI (Section 2).† In general, the corresponding aldehyde reacted with dipyrrylmethane to obtain the porphyrin precursors attached with protected amino chain ends, which were then subjected to hydrolysis to release the cationic end groups (Scheme S1†). The target porphyrins can be easily dissolved in water owing to their highly hydrophilic cationic side chains. Varying the number and structures of side chains aimed to tune the factors that may potentially impact their aggregation behaviors in aqueous solutions.

### Aqueous solubility and optical properties

The aggregation feature of porphyrins can be easily determined from their UV-vis absorbance and fluorescence emission as the aggregated porphyrins show characteristic spectroscopic properties compared to their monomeric state.^29^ Due to carrying highly hydrophilic positive end-charges on side chains, cationic porphyrins (Fig. 1) are usually very soluble and did not aggregate at all upon dissolving in water. For example, previously, we have reported a tetraphenyl porphyrin (TPP)-based photosensitizer (TPP 1, Fig. 1) with high water-solubility and enhanced type-I ROS generation capacity.^36^ As shown in Fig. S1a,† the absorption spectra of TPP 1 in water and a good solvent (methanol) are almost identical, indicating that there are no aggregates formed in water. Subsequently, fluorescence quenching was not observed for TPP 1 in water (Fig. S1b†). Unfortunately, biological polyanions (*e.g.*, ATP and PPi) could easily cause ACQ of TPP 1 in water (Fig. S2†), which is quite similar to that of other reported cationic TPP-type porphyrins with various types of side chains (linear or branched, Fig. 1).^29,30^ The ACQ of fluorescence of PSs could severely weaken the ROS generation capability, leading to poor performance for *in vivo* PDT of tumors, especially in a tumor environment where ATP is highly overexpressed.^7^

**Fig. 2:**
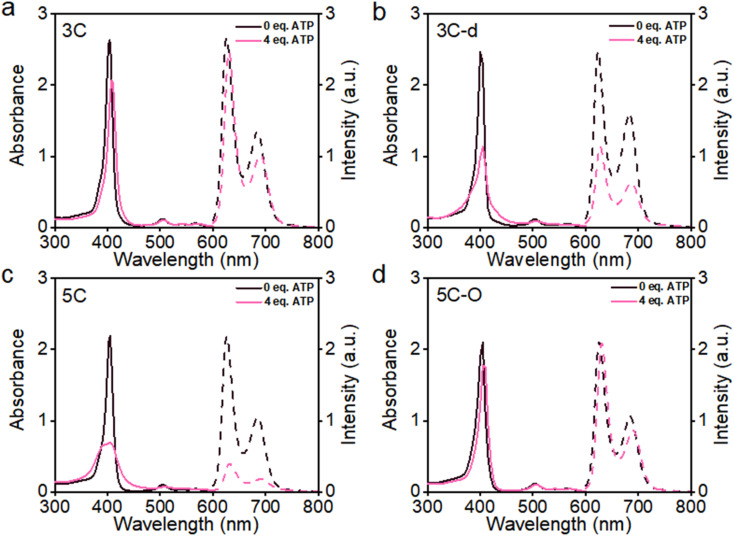
UV-vis absorption (solid line) and emission spectra (dashed line) of 3C (a) and less-branched 3C-d (b); 5C (c) and 5C–O with more hydrophilic chain spacers (d) in water before and after adding ATP as an aggregation inducer to quickly check the anti-ACQ behaviors to anions. *λ*_ex_ = 505 nm.

The flat conjugated plane of TPP-type porphyrins could facilitate the aggregation through π–π stacking when polyanions bring the porphyrin molecules close. Then the same type of sidechains was introduced to the 5′,15′-diphenyl porphyrins (DPP, 3C), to see if it could induce significant plane distortion to prevent anion-induced aggregation, as it is expected that a more distorted core plane might not be favored by π–π stacking of porphyrins. As expected, 3C is very soluble and does not self-aggregate in water like other cationic porphyrins (Fig. S3 and S4†). Moreover, the polyphosphate induced ACQ of fluorescence was not observed for 3C, which displayed only negligible changes in the UV-vis absorption and fluorescence emission spectra (Fig. 2a and S5†). With addition of sodium phytate which bears six phosphate groups per molecule, anti-ACQ properties were still retained by 3C (Fig. S5d†). The excellent anti-ACQ properties demonstrated by 3C to anions, especially to ATP, would greatly benefit the *in vivo* therapy of tumors, as ATP is highly over-expressed in tumor tissues.^37–39^

**Fig. 3:**
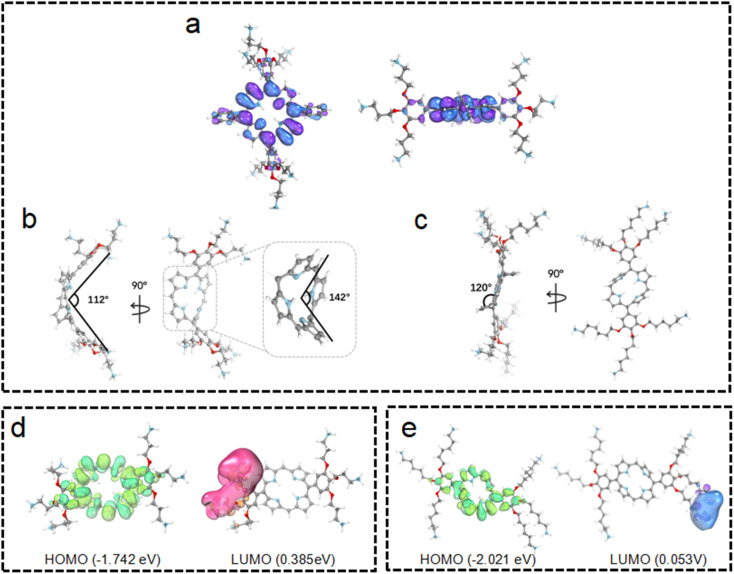
The quantum chemical-optimized geometries of TPP 1 (a), 3C (b) and 6C (c). The electron distributions of 3C (d) and 6C (e).

To obtain further insights into these observations, quantum chemical calculations were employed for the structures and electronic distributions of 3C and TPP 1. As shown in Fig. 3a and b, different from the plane structure of TPP 1, it can be seen that 3C adopts a saddle-shape distorted structure with a folding angle of 142°. Generally, the characteristic types of distortions in porphyrins are saddle, dome, ruffled and wave, while the saddle shape exhibits a higher degree of distortion of the porphyrin plane.^34^ The plane folding of 3C pushes the two bulky side chains to the back side of the folded plane with a dihedral angle of 112° (Fig. 3b). Unlike the tetraphenyl porphyrin TPP 1 with a flat plane structure, the significant plane distortion makes aggregation through π–π stacking unfavored by 3C. In addition, this bulky dendric side chain might further promote the plane distortion, and the orientation of bulky side chains would cause more steric effect that prevents porphyrins from stacking in aggregation.

The above-discussed results imply that the steric effect of bulky side chains plays a vital role in the distortion of diphenyl porphyrin. If this is the case, it would be expected to see that reducing the steric effect from side chains of 3C will weaken the deformation and enhance the flatness of the conjugated plane, which will lead to loss of resistance to the ACQ effect to a certain extent. To strengthen our hypothesis, two diphenyl porphyrin derivatives (3C-d and 3C-s, see structures in Fig. 1) were designed with less branched side chains to reduce the steric effect. The ATP titration experiments showed that 3C-d and 3C-s both have stronger tendency to aggregate in the presence of phosphates, due to decreasing the number of side chain branches (Fig. 2b and S6b†). Reasonably, the fluorescence of 3C-s with a single linear side chain was significantly quenched by ATP, while the fluorescence of 3C-d with bulkier side chains was much less quenched. Hence, this confirmed that the number of cationic branched side chains is critical to retain the ACQ resistance of diphenyl porphyrins.

It is well known that changing the overall hydrophobicity of the alkane chain spacer can affect the aggregation tendency of the molecules.^40^ The aggregation behaviors of cationic diphenyl porphyrins (4C, 5C and 6C, see structures in Fig. 1) with longer carbon chains than 3C were investigated. As shown in Fig. S6c,† with only one more carbon in each branched chain compared to 3C, porphyrin 4C retained the anti-ACQ properties to phosphates which is likely due to the similar hydrophilicity between 3C and 4C, while on further increasing the length of the carbon branched chain, 5C and 6C become significantly aggregated upon the addition of ATP (Fig. 2c and S6d†). In other words, with more hydrophobic chain spacers, the cationic diphenyl porphyrins tend to be more likely to aggregate in aqueous environments induced by phosphates. Therefore, it was confirmed that the hydrophobicity of chain spacers also plays a crucial role in determining their aggregation tendency in aqueous solutions. Quantum chemical simulations show that 6C has a chair-like wave shape, in which the dihedral angle between the two adjacent pyrrole subunits is about 120° (Fig. 3c). Despite having a wave-shape of pyrrole subunits, 6C is essentially planar, which may not destruct the π–π interactions between porphyrin rings.

For better understanding the structural difference, the electrochemical properties of 3C and 6C were further explored. Fig. 3d and e show the B3LYP orbitals of low-energy conformers of each cationic porphyrin. Herein, only the frontier orbitals, *i.e.*, the highest occupied molecular orbital (HOMO) and lowest unoccupied molecular orbital (LUMO) are considered. The HOMO energy of 6C (−2.021 eV) is lower than that of 3C (−1.742 eV), indicating that 3C has a much more distorted plane than 6C (Fig. 3d and e) which attenuates the overlapping of p-orbitals of the cyclic π-conjugated porphyrin core. These results implied that 3C molecules are less prone to aggregation than 6C from the perspective of energetics, as the destruction of the planarity and steric effect of peripheral substituents of highly saddled porphyrin are not favorable for the energetic stability of aggregation.^41^ The above observation is consistent with earlier reports suggesting that longer alkyl substituents play an important role in the process of aggregation.^42,43^ Collectively, the experimental results are further backed up by quantum chemical calculations, which provided promising mechanistic insights into the aggregation behaviors of cationic porphyrins.

Therefore, two key requirements can be drawn from the above for the design of cationic porphyrins with resistance to polyanion-induced ACQ in water, which are a DPP scaffold and branched side chains with proper hydrophilicity for inducing significant plane distortion of porphyrin. Conversely, if we perform structural modification on an ACQ porphyrin to increase the hydrophilicity of the chain spacer, will the porphyrin turn into non-aggregated as 3C? Porphyrin 5C was chosen as the analogous structure, and its side chain is easily accessible. The middle carbon of the chain spacer was replaced by an oxygen atom, which does not change the length of the chain but can increase the hydrophilicity. As expected, the analogous cationic porphyrin of 5C with oxygen in the chain spacer, 5C–O displays an anti-ACQ feature under the addition of ATP (Fig. 2d). This further confirmed the significant role of hydrophilicity of the chain spacer in regulating the polyanion-induced aggregation behaviors of cationic porphyrins. Conversely, it would be very easy to predict the aggregation tendency of the cationic porphyrin with a shorter carbon chain than 3C, such as DPP 2C with shorter side chains but enough hydrophilicity, which may decrease the steric effect on the conjugated plane. As shown in Fig. S6a,† it is clearly seen that 2C does not suffer from fluorescence quenching even when a high concentration of ATP was added, which further supported our proposed strategy.

### Aggregation behaviors in a physiological environment

**Fig. 4:**
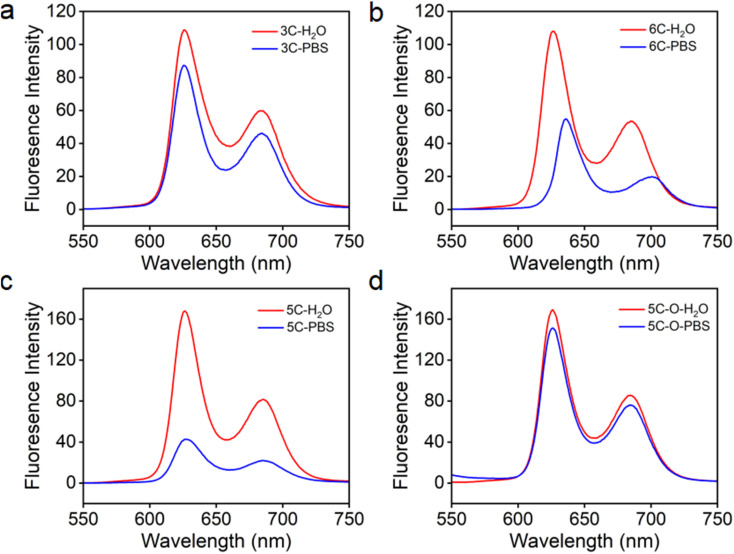
Fluorescence spectra of 3C (a), 6C (b), 5C (c), and 5C–O (d) in H_2_O (red) or PBS (blue).

It was reported that high ionic strength could cause cationic porphyrins to aggregate in water.^44^ As we know, inorganic ions (Na^+^, K^+^, PO_4_^3−^, *etc.*) and many other organic species exist at high concentrations under physiological conditions in humans. Therefore, cationic molecular PSs need to retain the anti-ACQ properties to high ion strength to tolerate the physiological environment for improved performance in subsequent biological applications. The ion-rich phosphate buffer saline (PBS, 10 mM) was used to simulate the physiological environment with high ionic strength to evaluate the fluorescence emission properties of the cationic porphyrins discussed above. As shown in Fig. S7a,† compared with that in water, significant fluorescence quenching in PBS was observed for TPP 1 which is not resistant to phosphate-induced-aggregation. 3C and 6C which bear cationic side chains with different spacer lengths exhibited almost identical fluorescence emission in water (Fig. 4a and b); however, their fluorescence emission showed significant difference in PBS. The emission of 3C in PBS is only slightly decreased, while the fluorescence of 6C in PBS is quenched by about 50% compared to that in water, which is closely related to the extent of plane distortion indicated by the computational simulations. Moreover, 5C and 5C–O also displayed similar fluorescence emission properties according to their aggregation behaviors in the presence of polyphosphates (Fig. 2c and d). The fluorescence emission of 5C–O in water and PBS was almost unchanged (Fig. 4d), while the fluorescence emission of 5C in PBS is sharply quenched (Fig. 4c). This was further supported by the emission of 2C and 4C in PBS (Fig. 7b and c) that DPP bearing longer branched sidechains with suitable hydrophilicity exhibits more robust anti-ACQ properties in a physiological environment. Uninhibited fluorescence emission of the porphyrins in the physiological environment would benefit diagnosis and PDT treatment of cancers.

### Evaluation of light-triggered ROS generation

**Fig. 5:**
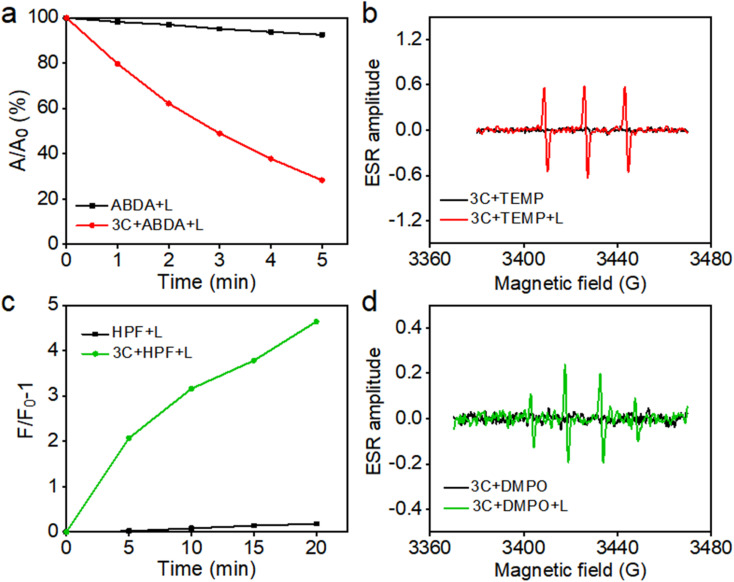
(a) Decomposition rates of ABDA with or without the presence of 3C with increasing illumination time. (b) ESR signals of 3C to detect the generation of ^1^O_2_ after irradiation using TEMP as a spin trapper. (c) Fluorescence changes of HPF with or without the presence of 3C with increasing illumination time. (d) ESR signals of 3C to detect the generation of OH˙ using DMPO as a spin trapper.

Based on their excellent water solubility and uninhibited fluorescence emission in a physiological environment, anti-ACQ porphyrins are expected to have good singlet oxygen (^1^O_2_) generating capability in water.^7^ Therefore, at first, 9,10-anthracenediyl-bis(methylene)-dimalonic acid (ABDA) was used to *in situ* evaluate the ^1^O_2_ production ability of 3C in water under photoirradiation. As shown in Fig. 5a, 3C exhibited excellent ^1^O_2_ generation ability in water as indicated by the decay curves of ABDA absorbances.

To further confirm ^1^O_2_ generation by 3C under light-irradiation, electron spin resonance (ESR) spectroscopy was employed by using 2,2,6,6-tetramethylpiperidine (TEMP) as the spin trapping agent for ^1^O_2_. As shown in Fig. 5b, only 3C produced a strong ESR signal of characteristic paramagnetic adducts from ^1^O_2_ upon photoirradiation, while no signal was captured in the dark. Previous work from us reported that TPP 1 is a dual PS which can produce both type-I and type-II ROS.^36^ Hence, we also switched to applying 5, 5-dimethyl-1-pyrroline-*N*-oxide (DMPO) as the spin trapper for any possible radicals produced by 3C. To our surprise, 3C clearly gave the characteristic ESR signal of OH˙ after light-irradiation; meanwhile, no other ESR signals were observed thereby confirming the OH˙ generation (Fig. 5d). Next, we used hydroxyphenyl fluorescein (HPF) as a specific fluorescent probe to further verify the generation of OH˙ (Fig. 5c). As expected, the fluorescence of HPF was enhanced on increasing the illumination time, confirming the OH˙ generation capability of 3C. This may be due to the strong intramolecular charge transfer between the porphyrin ring and the electron-donating oxygen linker in sidechains and cationic side chains, which enhances the generation capacity of type-I ROS.^45,46^ Cyclic voltammetry measurement was conducted to determine the redox potential of 3C. As shown in Fig. S8,† the reductive potential of 3C is determined to be −0.9 mV, which is less negative than that of the reported PSs which possess type-I or dual photodynamic pathways, and the anodic shifted reductive potential can facilitate the electron transfer between the PS and oxygen molecules or adjacent substrates.^3,11^ More importantly, TPP 1 showed severe aggregation and precipitation in PBS, leading to significant ROS inhibition, while anti-ACQ 3C exhibited much stronger ROS generation capability than TPP 1 (Fig. S9 and S10†) under identical conditions. This clearly indicates that the anti-ACQ properties of 3C offer a remarkable advantage for biological applications.

### Cellular uptake and *in vitro* PDT

**Fig. 6:**
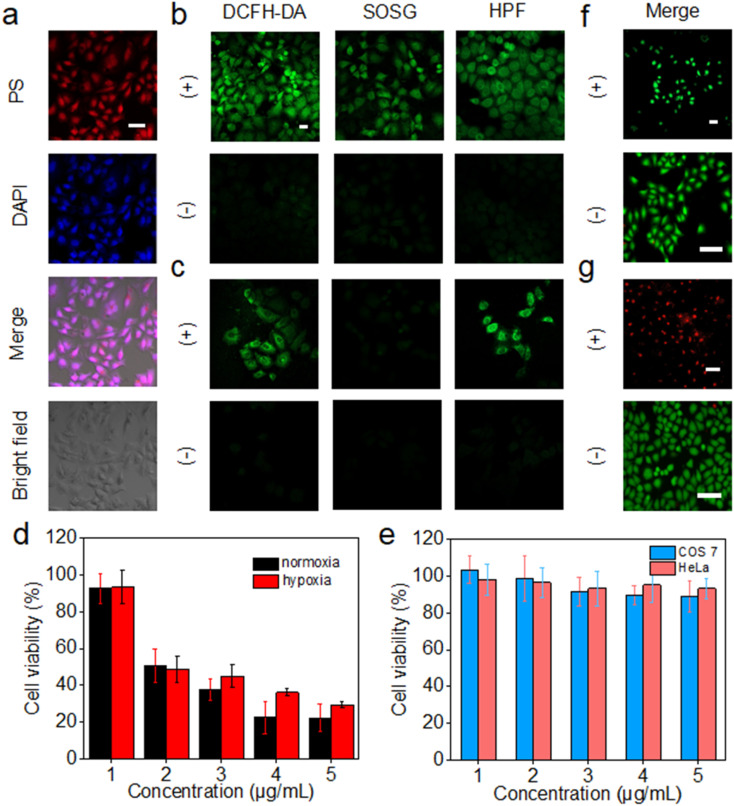
(a) Fluorescence images of HeLa cells incubated with 3C for 24 h. Detection of ROS, ^1^O_2_ and OH˙ in HeLa cells with DCFH-DA, SOSG and HPF, respectively, in normoxia (b) or hypoxia (c). (d) Phototoxicity toxicity of 3C on HeLa cells under normoxic (21% O_2_) and hypoxic (2% O_2_) conditions (*n* = 5). (e) Dark toxicity of 3C on COS 7 cells and HeLa cells under normoxia (*n* = 5). CLSM images of calcein AM/PI-stained HeLa cells incubated with 0 (f) or 5 μg mL^−1^ (g) 3C. [(+): 630 nm light irradiation, 50 mW cm^−2^, 20 min; (−): dark. Scale bar: 50 μm].

The intracellular uptake of 3C was evaluated by incubation with HeLa cells, followed by observation of the cells with a 4′,6-diamidino-2-phenylindole (DAPI) stained nucleus by confocal laser scanning microscopy (CLSM). 3C was easily taken up by HeLa cells at low concentration and located in the nucleus, indicated by the well-overlapped emission from DAPI and 3C (Fig. 6a). The positive charges of 3C might facilitate the cellular uptake and internalization in the nucleus.^47^ Based on the excellent ROS generating ability in water, we next routinely used 2′,7′-dichlorodihydrofluorescein diacetate (DCFH-DA) to probe the intracellular ROS generated by 3C under light-irradiation. As shown in Fig. 6b, strong green fluorescence was observed, suggesting that 3C internalized by cells has induced a considerable amount of ROS upon photoirradiation. Next, specific intracellular ROS probes, singlet oxygen sensor green (SOSG) and HPF, were used to identify the production of ^1^O_2_ and OH˙, respectively. It was obvious to see that bright green fluorescence emanated from the cells compared to that under dark conditions (Fig. 6b), indicating that both ^1^O_2_ and OH˙ were formed in cells under normoxia.

It is well known that the production of ^1^O_2_ through the type-II mechanism extremely relies on consumption of the O_2_ in cells, so it is hard for pure type-II PSs to achieve satisfactory effective *in vivo* PDT of solid tumors due to the hypoxic tumor microenvironment.^48^ As 3C can retain high efficacy in generating ^1^O_2_ and the less oxygen-dependent OH˙ due to its unique anti ACQ properties, it is expected to see 3C showing better *in vitro* PDT performance in hypoxia. Hence, HeLa cells were cultured in a hypoxic environment (2% O_2_) for evaluating the ROS generation efficacy of 3C in hypoxia. As shown in Fig. 6c, green fluorescence was emitted from DCFH-DA in HeLa cells after photoirradiation, while only a negligible fluorescence signal from SOSG was observed, indicating that the dominant ROS generated in hypoxia was not ^1^O_2_. Taken together, these results demonstrated that the type-I photodynamic mechanism was preferred for the ROS generation in hypoxia. The green fluorescence emanated from HPF has further confirmed the intracellular formation of OH˙ which was generated by the type-I photodynamic pathway (Fig. 6c). Therefore, based on the favorable results discussed above, we could expect that 3C possesses good PDT efficacy in both normoxia and hypoxia.

Next, we evaluated the cell cytotoxicity and *in vitro* PDT efficacy of 3C using the standard 3-(4,5-dimethylthiazol-2-yl)-2,5-diphenyltetrazolium bromide (MTT) assay. As shown in Fig. 6e, after incubation with COS 7 cells and HeLa cells for 24 h, respectively, low dark toxicity to normal cells and cancer cells indicated the good biocompatibility of 3C. Furthermore, 3C displayed strong cytotoxicity towards HeLa cells upon photoirradiation (50 mW cm^−2^) with a low IC_50_ value of 2.54 μM owing to its effective intracellular ROS generation (Fig. 6d). The phototoxicity of 3C to HeLa cells under hypoxic conditions was also evaluated, which is shown in Fig. 6d with an IC_50_ value of 3.04 μM. The approximate IC_50_ values for HeLa cells in normoxia and hypoxia indicated the effective *in vitro* PDT of 3C even in a hypoxic environment, which results from the good capability of producing the highly toxic and less oxygen-dependent OH˙ of 3C.

Next, calcein-AM and propidium iodide (PI) assay was performed to visualize the cancer cell-inhibiting capability of 3C by tracking the green fluorescence (calcein-AM) from living cells and red fluorescence (PI) from dead cells. As shown in Fig. 6f and g, only red emission of PI was observed from the HeLa cells treated with 5 μg mL^−1^ of 3C after illumination. In contrast, with only illumination but without incubation with 3C, just the green fluorescence of calcein-AM was emitted from the cells, indicating the negative impact of light on causing the death of cells. Taken together, with the fact that 3C exhibited strong phototoxicity and negligible dark cytotoxicity to cancer cells, precise and effective accumulation of 3C at the tumor site would allow the highly effective *in vivo* PDT of cancer.

### *In vivo* tumor NIR imaging and therapy

**Fig. 7:**
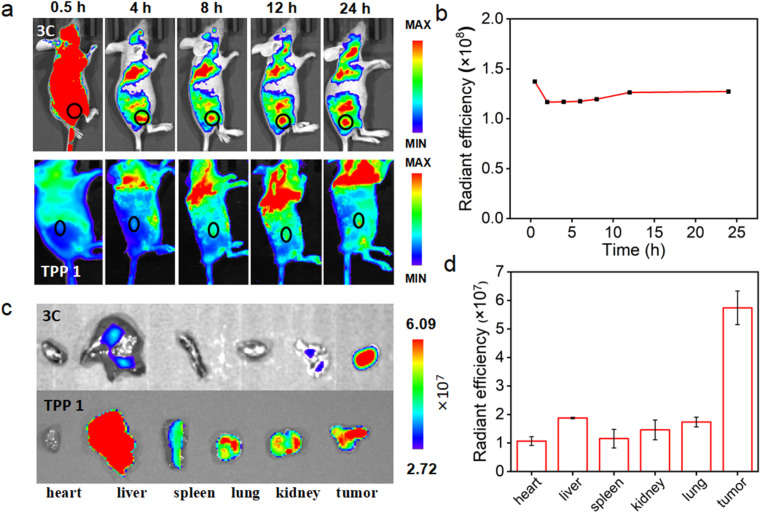
(a) The fluorescence images tracking the distribution and delivery of 3C and TPP 1 to the tumor site of the mouse over 24 hours. Black circles indicate the location of the tumor. (b) Fluorescence intensities at the tumor site of the mouse injected with 3C. (c) Fluorescence images of the *ex vivo* organs harvested at 24 hours after injection of 3C and TPP 1. (d) Fluorescence intensities of the *ex vivo* organs of the mice injected with 3C. Error bars represent the standard deviation (*n* = 3).

Encouraged by the excellent *in vitro* PDT performance of 3C, *in vivo* distribution of 3C was evaluated in 4T1 tumor-bearing BALB/c mice prior to conducting the *in vivo* PDT study. As 3C is highly soluble in water, the use of a hydrophilic organic solvent or encapsulation agent for assisting the dissolution of PSs, like that reported previously was avoided.^3,45,49^ Hence, we used a low dose of PS by diluting an aqueous stock solution of 3C directly in 100 μL of saline for intravenous injection through the tail vein of mice (1 mg kg^−1^).^7^ TPP 1 was injected into mice using the same procedure as 3C to serve as a control. As shown in Fig. 7a, after the injection of 3C, strong fluorescence was emitted from the body of the mice and started accumulating at tumor sites 2 hours after injection. Since the 6th hour post injection, the tumor site emitted much stronger fluorescence than the area surrounding the tumor, indicating the effective accumulation of 3C at the tumor site, which is presumably due to its excellent aqueous solubility and long blood circulation time.^50–52^ Moreover, the time-dependent fluorescence intensities at the tumor site did not decrease 24 h post injection, showing the excellent retention and long half-life of 3C in the tumor (Fig. 7a and b), while the half-life of Ce6 was only reported to be 3.69 h.^53^ Some porphyrin derivatives have shown tumor targeting ability, and especially those with low lipophilicity can further reduce non-specific accumulation in organs.^53,54^ In addition, the high concentration of extracellular ATP in tumor tissue may contribute to the retention of 3C through the electrostatic interaction between positive charges of 3C and negative charges of ATP. However, no significant accumulation of TPP 1 was observed at the tumor site after 24 hours of blood circulation in mice (Fig. 7a). This may be due to the fact that TPP 1 is easily induced to aggregate by polyanions in the blood and thus hard to target and accumulate at the tumor site. At 24 h post injection of 3C and TPP 1, the organs and tumors were harvested for *ex vivo* fluorescence analysis. As shown in Fig. 7c, approximate fluorescence emission was observed at the tumor site and organs, which indicated the non-specific targeting ability of TPP 1. While for the mice injected with 3C, much stronger fluorescence was recorded in tumors than the organs, which has further confirmed the effective accumulation and high retention of 3C in the tumor (Fig. 7d).

**Fig. 8:**
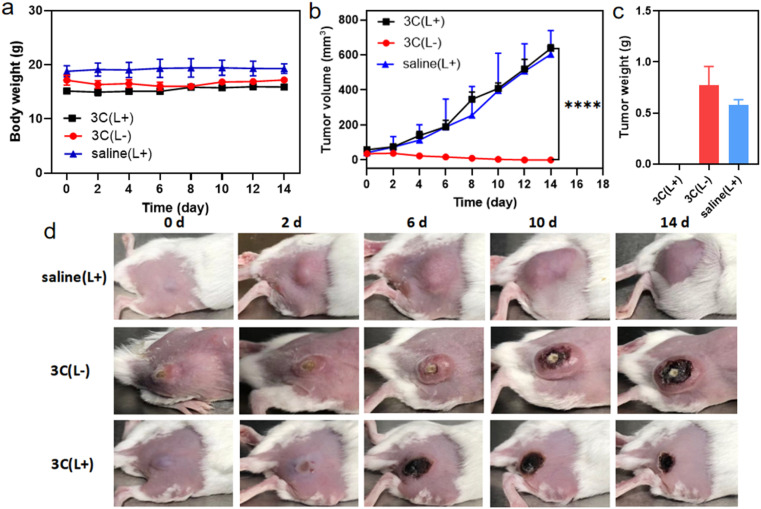
(a) Body weights of the mice during the observation. (b) Tumor growth profiles during the observation. *P < 0.05, **P < 0.01, ***P < 0.001 and ****P < 0.0001 (*t*-test). (c) Averaged weights of the dissected tumor of mice in different groups. Error bars represent the standard deviation (*n* = 3). (d) Representative photos of tumor-bearing mice during PDT treatment. (L+): laser irradiation and (L-): no laser irradiation.

Next, the *in vivo* therapeutic efficacy of 3C was evaluated in 4T1 tumor-bearing mice. The mice were divided into 3 groups and treated as follows: saline solution injection plus photoirradiation; 3C saline solution injection only; 3C saline solution injection plus photoirradiation (635 nm, 0.5 W cm^−2^). The photoirradiation was performed on the tumor sites for 10 min at 24 h post injection. Then, the tumor volumes and body weights of the mice were measured every other day to track the *in vivo* therapeutic effect of 3C on solid tumors. The weights of the mice changed negligibly in all the three groups (Fig. 8a), suggesting the excellent biocompatibility of 3C. A few days after the treatment, tumors in the therapeutic group completely disappeared without relapse, while complete tumor ablation was not achieved in the two control groups (Fig. 8b–d and S11†). At the end, H&E-staining was carried out to further examine the biosafety of the treatment in each group. There was no noticeable pathological change observed in the harvested organs (heart, liver, spleen, lung, and kidneys) from the mice in all groups (Fig. S12†). These results have demonstrated that 3C is a safe and effective molecular PS for PDT of solid tumors, as a result of its excellent anti-ACQ properties in an aqueous medium.

## Conclusions

In summary, we have developed a strategy for designing molecular PSs with remarkable solubility and anti-ACQ properties in an aqueous media to improve the fluorescence emission and *in vivo* PDT efficacy. The chemical structures of porphyrins are characteristic with a DPP core and various cationic side chains on the phenyl rings. Based on experimental data and computational simulations, it was found that the cationic branched side chains with proper hydrophilicity in the chain spacer exhibit an enhanced steric effect on the conjugated plane of porphyrin and lead to significant plane distortion, which are unfavored for aggregate formation *via* π–π interactions. It is worth emphasizing that the anti-ACQ properties of the porphyrins could be retained even in the presence of polyanions or under high ionic strength, leading to unsuppressed fluorescence emission and excellent ROS generation capability (^1^O_2_ and OH˙). Moreover, the *in vivo* delivery efficacy of anti-ACQ porphyrin was also significantly enhanced compared to that of ACQ porphyrin, displaying precise and efficient tumor accumulation and retention through whole-body blood circulation, leading to great antitumor efficacy and complete tumor ablation. This work provides a new strategy and deep insights into designing non-AIE type PSs to boost the fluorescence and *in vivo* tumor PDT performance.

## Data availability

All the data was included in the ESI.†

## Author contributions

H. Sun synthesized the molecules, conducted the measurements and data analysis and wrote the initial manuscript. L. Li synthesized compound d-3C, conducted the measurements and data analysis. R. Guo performed some of the cell and animal experiments. Z. Wang conducted the computational simulations and wrote the method. Y. Guo synthesized compound s-3C. F. Song contributed to project administration and funding. Z. Li contributed to the draft and project administration, funding, conceptualization, writing, reviewing & editing the manuscript. All authors discussed and commentated on the manuscript.

## Conflicts of interest

The authors declare no competing financial interest.

## Supplementary Material

SC-015-D3SC05041F-s001
