# Supplementary material for: Suppressing ACQ of molecular photosensitizers by distorting the conjugated-plane for enhanced tumor photodynamic therapy
Source: Chem Sci. 2023 Dec 12;15(3):940–52. doi: 10.1039/d3sc05041f (PMC10793593; doi:10.1039/d3sc05041f)
Supplement: SC-015-D3SC05041F-s001 [file SC-015-D3SC05041F-s001.pdf]

Electronic Supplementary Material (ESI) for Chemical Science.
This journal is © The Royal Society of Chemistry 2023

Supporting Information

Suppressing ACQ of Molecular Photosensitizers by Distorting the Conjugated-Plane for Enhanced Tumor Photodynamic Therapy

Han Sun†, Lukun Li†, Ruihua Guo†, Zhe Wang#,* Yanhui Guo†, Zhiliang Li†,*, Fengling Song†,*

† Institute of Molecular Science and Engineering, Institute of Frontier and Interdisciplinary Science, Shandong University, Qingdao, Shandong 266237, China.

# Department of Materials science and engineering, Hainan University, Haikou, Hainan 570228, China.

1.General information

1.1 Materials and Instruments.

Dichloromethane (DCM), dimethyl sulfoxide (DMSO), acetone, methanol, concentrated hydrochloric acid (HCl), trifluoroacetic acid (TFA), triethylamine and potassium carbonate were purchased from Sinopharm Chemical Reagent Co., Ltd. Pyrrole, tert-butyl-3-bromopropylcarbamate, 2,3-dichloro-5,6-dicyano-1,4-benzoquinone (DDQ), tosyl chloride, 3,4,5-trihydroxybenzaldehyde, 2,2,6,6-tetramethylpiperidine (TEMP) and 5,5-dimethyl-1-pyrroline-N-oxide (DMPO) were purchased from Shanghai Macklin Biochemical Co., Ltd. Dioxane, paraformaldehyde, di(tert-butyl)carbonate were purchased from Shanghai Aladdin Biochemical Technology Co., Ltd. 3,5-dihydroxybenzaldehyde, 4-hydroxybenzaldehyde, 4-amino-1-butanol, tert-butyl-N-(2-bromoethyl)carbamate, 6-amino-1-hexanol, 5-amino-1-pentanol were purchased from Anhui Zesheng Technology Co., Ltd. The organic solvents were purchased from commercial suppliers and used as received. Absorption spectra of the samples were measured on the HITACH UH5300 spectrophotometer. Fluorescence spectra were made on HITACH F-4700 spectrophotometer. NMR spectra were recorded with nuclear magnetic resonance spectrometer (Bruker AVANCE NEO). High-resolution mass spectrometry experiments were made on Bruker impact HD. Dynamic light scattering (DLS) size measurements were performed using Malvern Nano ZSE. Fluorescence images of cells were performed with Zeiss LSM 900. Cell viability test was performed on Thermo Scientific Multiskan FC. *In vivo* imaging was made on an IVIS Spectrum imaging system (Perkinelmer). Photoirradiation was conducted by a LED light (630 nm) or laser (635 nm).

1.2 ROS Detection with electron spin resonance (ESR)

DMPO was dispersed in water at a concentration of 400 mM for trapping the formation of OH·. TEMP was used as trapping agent for $^1O_2$. The tested porphyrin samples were dissolved in water at a concentration of 10 μM. The ESR spectra of the mixture were recorded before and after illumination by a 630 nm LED light (10 min, 50 mW/cm^2).

1.3 $^1O_2$ detection

9,10-anthracenediyl-bis(methylene)-dimalonic acid (ABDA) was used for probing the generation of $^1O_2$ under light irradiation. A 2mL of mixed solution containing ABDA (100 μM) and porphyrin (10 μM) was placed in a cuvette and irradiated with a 630 nm LED light (50 mW/cm^2), then the absorbance of ABDA at 377 nm was recorded with a UV-Vis absorption spectrophotometer.

1.4 OH· detection

Hydroxyphenyl fluorescein (HPF) was used to monitor the production of OH·. HPF (25 μM) was added to the aqueous solution of **3C** (10 μM), followed by placing the above solution under laser irradiation for 10 min (635 nm, 500 mW/cm^2). The fluorescence signal of HPF monitored by a fluorescence spectrophotometer raised after irradiation, indicating the presence of OH·.

1.5 *In vitro* cellular uptake

Cells were incubated with **3C** at 37 ℃, under 5% CO_2 atmosphere for 12 h. After incubation, the HeLa cells were rinsed with PBS, fixed with paraformaldehyde, stained with DAPI for observation by CLSM.

1.6 *In vitro* cytotoxicity

Cells were seeded in 96-well plates and incubated for 24 h under normoxic and hypoxic atmosphere, respectively. Then, the concentrations of added **3C** solutions were varied for cytotoxicity study. The plates were irradiated with a LED light for 20 min (630 nm,50 mW/cm^2) or kept in dark after incubating with **3C** for another 12 h in normoxia or hypoxia. After that, the cells were cultured for about 8-12 h and then treated with 20 μL of MTT in PBS (5 mg/mL). After another 4 h of incubation, DMSO was added. Finally, the corresponding absorbance at 620 nm was measured using a microplate reader.

1.7 Intracellular ROS detection

2′, 7′-dichlorodihydrofluorescein diacetate (DCFH-DA) was used as indicators for overall ROS generation, which can be converted to DCF and emit green fluorescence at 525 nm when oxidized by ROS. HeLa cells were seeded in cell culture dish and incubated overnight in normoxia or hypoxia condition, followed by adding **3C** solution (1 μg/mL) and incubating another 12 h under the same conditions. After rinse with PBS, the cells were incubated with 10 μM DCFH-DA for 20 min. The treated cells were exposed to irradiation for 10 min with a 635 nm laser (500 mW/cm^2). After irradiation, the fluorescence imaging was taken on CLSM.

1.8 Intracellular $^1O_2$ detection

The intracellular $^1O_2$ detection followed the same procedure as detailed in 1.7 for ROS by using singlet oxygen sensor green (SOSG) as the indicator instead of using DCFH-DA. The working concentration of SOSG is chosen at 5 μM.

1.9 Intracellular OH· detection

Hydroxyphenyl fluorescein (HPF) was used to detect the production of OH·. The testing process of OH· is the same as that detailed in 1.7 for detecting ROS. The working concentration of SOSG is chosen at 50 μM. Then, the cell images were collected on a CLSM.

1.10 Calcein-AM/PI staining of HeLa cells

The HeLa cells were seeded on 20 mm confocal dishes and incubated overnight. Then the cells were incubated with different concentrations of **3C** solutions (0,5 μg/mL) for 12 h. After that, the confocal dishes were irradiated with a LED light for 20 min or kept in dark. Then, the cells were stained with a PBS solution containing 2 μM Calcein-AM and 8 μM PI. Confocal images were taken to observe the survival and death of cells.

1.11 Cyclic voltammetry measurement

The cyclic voltammetry measurement of **3C** (20 μM) was conducted in a three-electrode system. H_2O was selected as a supporting electrolyte. Glassy carbon, Ag/AgCl, Pt wire were employed as work electrode, reference electrode and counter electrode respectively, with a scan rate of 100 $mV{\cdot}s^{-1}$.

1.12 Computational detail

Quantum chemical calculations were used to determine the geometry and electronic structure of porphyrin molecules. The calculations were performed with ORCA 5.0.3[1] All calculated structures were verified as true minima by the absence of negative eigenvalues in the harmonic vibrational frequency analysis. Tighter than default convergence criteria (tightopt), grid values (grid5, finalgrid6) were chosen for both the optimization of the structural parameters and the scf (tightscf). The geometry optimizations were performed at the B3LYP-D3/def2-TSVP level of theory[2, 3]. The RIJCOSX approximation and the related auxiliary basis set def2/J were used to speed up the calculations[4, 5]. Molecular orbitals and optimized geometries were visualized with IBOView[6]. The water solvent effects are implicitly considered by the universal solvation model[7].

1.13 Animal model

All experiments related to animals were implemented in accordance to the protocols approved by the local Ethical Committee in compliance with the China Animal Management Regulations. The female BALB/c mice were purchased from Jinan Pengyue Laboratory Animal Breeding Co. Ltd. Cell suspension was prepared by subculture and amplification of 4T1 cell line, and the suspension was inoculated subcutaneously with 5.0×10^7 cells mL^{-1} in BALB/c mice.

1.14 *In vivo* fluorescence imaging

4T1 Tumor-bearing BALB/c mice were intravenously injected with **3C** or **TPP 1** for *in vivo* imaging. The distribution of drugs in mice was recorded with an IVIS spectrum imaging system. Then, at 24 h postinjection, the mice were sacrificed, and their harvested organs (liver, lung, spleen, kidney, and heart) and tumors were imaged under the imaging system.

1.15 *In vivo* photodynamic therapy (PDT)

The mice were divided into three groups randomly for treatment. 1) saline solution injection only (control group); 2) **3C** aqueous solution injection only; 3) **3C** aqueous solution plus laser irradiation (experimental group). **3C** saline solutions (1 μmol/kg) were injected via tail vein of the mice at 0, 2, 4 day. After a 24-hour cycle, control group and experimental group were irradiated with a 635 nm laser for 10 min (500 mW/cm^2). The tumor size (tumor size = width × width × length / 2.) and body weights of the mice were measured and recorded for 2 weeks. Then, tumors and main organs (liver, lung, spleen, kidney, and heart) were dissected from the mice for histological analysis by hematoxylin-eosin (H&E) staining.

2. General Synthesis Methods

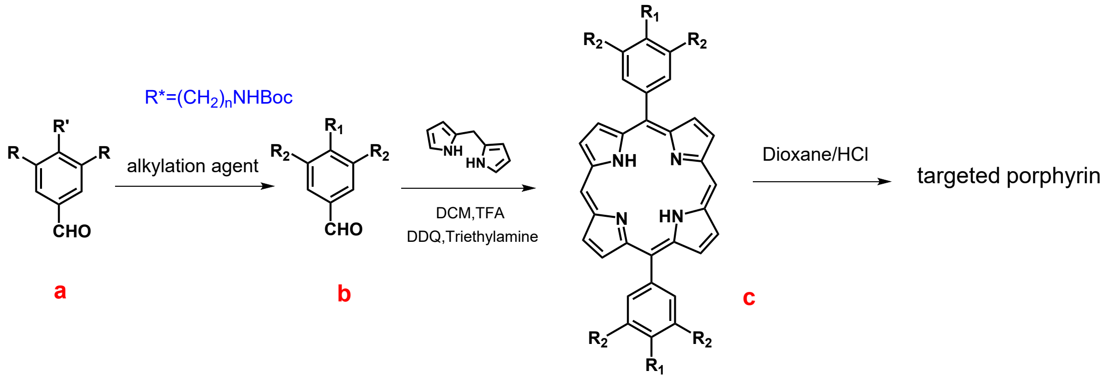

Scheme S1. General synthetic route of targeted porphyrins.

The synthesis methods of the alkylation agent and compound b in this paper refer to literatures.[8, 9]

General procedure **1** for synthesis of compound **b**

The hydroxybenzaldehyde and alkylation agent was dissolved in acetone or DMF, respectively. Then, the two solutions were mixed and stirred at room temperature under a nitrogen atmosphere for 20 min, followed by adding potassium carbonate. The resulting mixture was refluxed and monitored by TLC until the reaction was finished. Then, the mixture was diluted with dichloromethane (DCM). The organic phase was dried over anhydrous Na_2SO_4 and evaporated under reduced pressure. The crude product was then purified by column chromatography with silica gel.

di-tert-butyl(((2-(2-((tert-butoxycarbonyl)amino)ethoxy)-5-formyl-1,3-phenylene)bis(oxy))bis(ethane-2,1-diyl))dicarbamate **(b_1)**

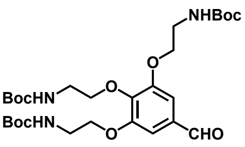

b_1 was synthesized according to the general procedure 1 using 3,4,5-trihydroxybenzaldehyde (0.4 mmol), alkylation agent (1.4 mmol), potassium carbonate (1.4 mmol). The mixture refluxed in acetone until the reaction is complete. The crude material was purified by column chromatography with silica gel and dichloromethane / ethyl acetate 3:1 as the eluent.

di-tert-butyl(((2-(3-((tert-butoxycarbonyl)amino)propoxy)-5-formyl-1,3-phenylene)bis(oxy))bis(propane-3,1-diyl))dicarbamate **(b_2)**

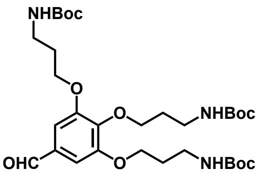

b_2 was synthesized according to the general procedure 1 using 3,4,5-trihydroxybenzaldehyde (0.4 mmol), alkylation agent (1.4 mmol), potassium carbonate (1.4 mmol). The mixture refluxed in acetone until the reaction is complete. The crude material was purified by column chromatography with silica gel and dichloromethane / ethyl acetate 3:1 as the eluent. ^1H NMR (600 MHz, $CDCl_3$) δ 9.84 (s, 1H), 7.11 (s, 2H), 5.35 (s, 1H), 5.09 (s, 2H), 4.16 – 4.13 (m, 6H), 3.40 (t, J = 6.7 Hz, 4H), 3.35 (t, J = 6.7 Hz, 2H), 2.06 – 2.04 (m, 6H), 1.45 (s, 9H), 1.44

(s, 18H).; ^{13}C NMR (150 MHz, $CDCl_3$) δ 190.94, 156.15, 153.03, 142.87, 131.80, 107.64, 79.24, 79.13, 71.81, 67.21, 38.38, 38.06, 30.13, 29.66, 28.50, 28.45. Molecular Weight = 625.76 g/mol; - Molecular Formula = $C_{31}H_{51}N_3O_{10}$

di-tert-butyl (((5-formyl-1,3-phenylene)bis(oxy))bis(propane-3,1-diyl))dicarbamate **(b_3)**

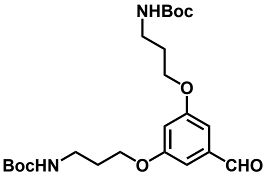

b_3 was synthesized according to the general procedure 1 using 3,5-dihydroxybenzaldehyde (0.4 mmol), alkylation agent (1 mmol), potassium carbonate (1 mmol). The mixture refluxed in acetone until the reaction is complete. The crude material was purified by column chromatography with silica gel and dichloromethane / ethyl acetate 5:1 as the eluent. ^{1}H NMR (600 MHz, $CDCl_3$) δ 9.89 (s, 1H), 7.00 (d, J = 2.3 Hz, 2H), 6.70 (t, J = 2.3 Hz, 1H), 4.73 (s, 2H), 4.05 (t, J = 6.1 Hz, 4H), 3.35 – 3.32 (m, 4H), 2.02 – 1.98 (m, 4H), 1.44 (s, 18H). ^{13}C NMR (150 MHz, $CDCl_3$) δ 191.85, 160.54, 156.21, 138.41, 108.00, 107.75, 78.97, 66.08, 37.70, 29.56, 28.44. Molecular Weight = 452.55 g/mol; - Molecular Formula = $C_{23}H_{36}N_2O_7$

tert-butyl (3-(4-formylphenoxy)propyl)carbamate **(b_4)**

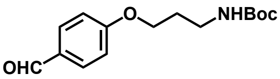

b_4 was synthesized according to the general procedure 1 using 4-hydroxybenzaldehyde (0.4 mmol), alkylation agent (0.6 mmol), potassium carbonate (0.6 mmol). The mixture refluxed in acetone until the reaction is complete. The crude material was purified by column chromatography with silica gel and dichloromethane / ethyl acetate 6:1 as the eluent.

di-tert-butyl(((2-(4-((tert-butoxycarbonyl)amino)butoxy)-5-formyl-1,3-phenylene)bis(oxy))bis(butane-4,1-diyl))dicarbamate **(b_5)**

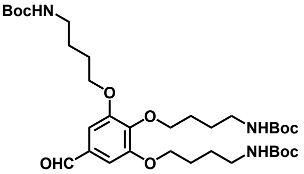

b_5 was synthesized according to the general procedure 1 using 3,4,5-trihydroxybenzaldehyde (0.4 mmol), alkylation agent (1.4 mmol), potassium carbonate (1.4 mmol). The mixture refluxed in DMF under 80 ℃ until the reaction is complete. The crude material was purified by column chromatography with silica gel and dichloromethane / ethyl acetate 3:1 as the eluent. ^{1}H NMR (600 MHz, $CDCl_3$) δ 9.83 (s, 1H), 7.08 (s, 2H), 4.09 – 4.05 (m, 6H), 3.20 (m, 6H), 1.89 – 1.84 (m, 4H), 1.79 (dd, J = 8.7, 5.5 Hz, 2H), 1.73 – 1.66 (m, 6H), 1.44 (s, 20H), 1.44 (s, 7H). ^{13}C NMR (151 MHz, MeOD) δ 191.78, 157.14, 153.30, 143.12, 132.01, 107.44, 103.04, 78.46, 72.85, 72.56, 68.49, 68.30, 55.57, 39.18, 27.45, 27.41, 26.39, 26.30, 15.42. Molecular Weight = 667.84 g/mol; - Molecular Formula = $C_{34}H_{57}N_3O_{10}$

di-tert-butyl(((2-((5-((tert-butoxycarbonyl)amino)pentyl)oxy)-5-formyl-1,3-phenylene)bis(oxy))bis(pentane-5,1-diyl))dicarbamate **(b_6)**

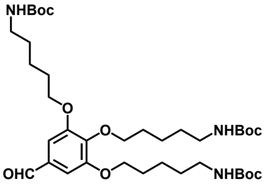

b_6 was synthesized according to the general procedure 1 using 3,4,5-trihydroxybenzaldehyde (0.4 mmol), alkylation agent (1.4 mmol), potassium carbonate (1.4 mmol). The mixture refluxed in DMF under 80 ℃ until the reaction is complete. The crude material was purified by column chromatography with silica gel and dichloromethane / ethyl acetate 3:1 as the eluent. ^{1}H NMR (600 MHz, $CDCl_3$) δ 9.83 (s, 1H), 7.08 (s, 2H), 4.06 – 4.02 (m, 6H), 3.16 – 3.12 (m, 6H), 1.85 (dd, J = 14.3, 6.5 Hz, 6H), 1.56 – 1.51 (m, 12H), 1.44 (s, 27H). ^{13}C NMR (151 MHz, $CDCl_3$) δ 191.20, 156.09, 156.07, 153.38, 143.58, 131.57, 107.85, 98.93, 79.12, 73.31, 68.99, 67.40, 53.43, 29.97, 29.88, 29.86, 28.93, 28.47, 28.45, 23.40, 23.35. Molecular Weight = 709.92 g/mol; - Molecular Formula = $C_{37}H_{63}N_3O_{10}$

di-tert-butyl(((2-((6-((tert-butoxycarbonyl)amino)hexyl)oxy)-5-formyl-1,3-phenylene)bis(oxy))bis(hexane-6,1-diyl))dicarbamate **(b_7)**

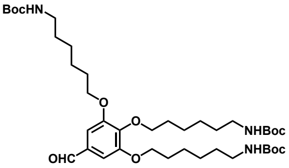

b_7 was synthesized according to the general procedure 1 using 3,4,5-trihydroxybenzaldehyde (0.4 mmol), alkylation agent (1.4 mmol), potassium carbonate (1.4 mmol). The mixture refluxed in DMF under 80 ℃ until the reaction is complete. The crude material was purified by column chromatography with silica gel and dichloromethane / ethyl acetate 3:1 as the eluent. ^{1}H NMR (600 MHz, $CDCl_3$) δ 9.83 (s, 1H), 7.08 (s, 2H), 4.59 (s, 3H), 4.05 – 4.02 (m, 6H), 3.12 – 3.10 (m, 6H), 1.84 – 1.82 (m, 4H), 1.76 – 1.74 (m, 2H), 1.52 – 1.50 (m, 12H), 1.44 (s, 27H), 1.40 – 1.38 (m, 6H). ^{13}C NMR (150 MHz, $CDCl_3$) δ 191.25, 156.06, 153.43, 143.70, 131.52, 107.86, 79.09, 73.44, 69.06, 40.63, 30.23, 30.11, 30.01, 29.17, 29.12, 28.44, 26.67, 26.55, 26.43, 25.87, 25.80, 25.75. Molecular Weight = 752.00 g/mol; - Molecular Formula = $C_{40}H_{69}N_3O_{10}$

di-tert-butyl(((((2-(2-(2-((tert-butoxycarbonyl)amino)ethoxy)ethoxy)-5-formyl-1,3-phenylene)bis(oxy))bis(ethane-2,1-diyl))bis(oxy))bis(ethane-2,1-diyl))dicarbamate **(b_8)**

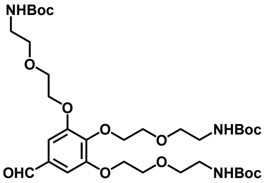

b_8 was synthesized according to the general procedure 1 using 3,4,5-trihydroxybenzaldehyde (0.4 mmol), alkylation agent (1.4 mmol), potassium carbonate (1.4 mmol). The mixture refluxed in DMF under 80 ℃ until the reaction is complete. The crude material was purified by column chromatography with silica gel and dichloromethane / ethyl acetate 3:1 as the eluent. ^{1}H NMR (600 MHz, $CDCl_3$) δ 9.84 (s, 1H), 7.16 (s, 2H), 4.32 – 4.27 (m, 2H), 4.26 – 4.21 (m, 4H), 3.87 – 3.83 (m, 4H), 3.81 – 3.77 (m, 2H), 3.60 (dt, J = 11.4, 5.0 Hz, 6H), 3.36 – 3.28 (m, 6H), 1.43 (s, 27H).

General procedure **2** for synthesis of compound **c**.

The compound **c** were synthesized according to the well-developed method. Dipyrromethane (0.16 mmol) and the corresponding substituted aldehydes (b_1-b_8, 0.16 mmol) were dissolved in DCM (20 mL) and stirred in dark at room temperature under a nitrogen atmosphere for 20 min. Trifluoroacetic acid (TFA, 0.256 mmol) was added subsequently. The mixture was stirred overnight and then DDQ (0.256 mmol) was added. After stirring for another hour, triethylamine (40 μL) was added to quench TFA. The mixture was evaporated under reduced pressure, and the residue was purified by column chromatography with silica gel using dichloromethane/ methanol (40:1) as the eluent. The solid obtained from above was dissolved in dioxane (4 mL) followed by the addition of concentrated HCl solution (2 mL) at 0 ℃. Then, the resulting mixture was shielded from light and stirred overnight at room temperature. The reaction mixture was added dropwise into cold acetone (20 mL) for precipitation. The obtained green precipitates were then centrifugated and redissolved into pH=3-4 deionized water. The final product was obtained as a green solid after freeze-drying. Finally, the green solid was dissolved in pH=2-3 deionized water and adjusted to a concentration of 2 mmol/L as stock solution.

Compound **2C**

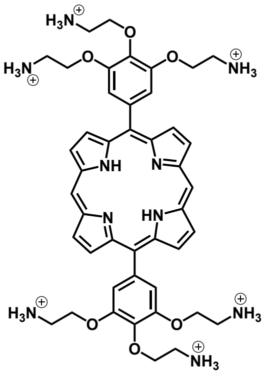

^{1}H NMR (600 MHz, CD_3OD+D_2O) δ 11.15 (s, 2H), 9.82 (dd, J = 4.8, 1.6 Hz, 4H), 9.36 (dd, J = 4.8, 1.3 Hz, 4H), 7.92 (s, 4H), 4.64 (t, J = 4.9 Hz, 4H), 4.59 (t, J = 5.2 Hz, 8H), 3.60 (m, 12H). ^{13}C NMR (150 MHz, CD_3OD+D_2O) δ 152.26, 147.40, 145.36, 138.52, 138.03, 133.42, 132.39, 121.43, 117.85, 108.41, 71.41, 67.10, 41.28, 40.52. HRMS: m/z; $[M]^{2+}$, calcd for $C_{44}H_{54}N_{10}O_6^{2+}$: 409.2109; found: 409.2116.

Compound **3C**

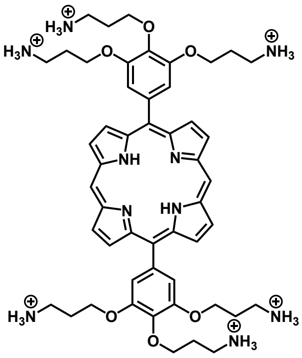

^{1}H NMR (600 MHz, CD_3OD+D_2O) δ 10.87 (s, 2H), 9.76 (d, J = 4.6 Hz, 4H), 9.30 (d, J = 4.6 Hz, 4H), 7.78 (s, 4H), 4.57 (t, J = 5.9 Hz, 4H), 4.43 (t, J = 6.1 Hz, 8H), 3.53 (t, J = 7.4 Hz, 4H), 3.35 – 3.34 (m, 8H), 2.43 – 2.36 (m, 12H). ^{13}C NMR (150 MHz, CD_3OD+D_2O) δ 151.24, 145.69, 142.98, 137.54, 135.76, 131.25, 130.63, 120.79, 117.17, 107.11, 71.94, 66.74, 37.88, 37.22, 27.53, 26.81. HRMS: m/z; $[M]^{+}$, calcd for $C_{50}H_{68}N_{10}O_6^{4+}$: 226.1325; found: 226.1324.

Compound **3C-d**

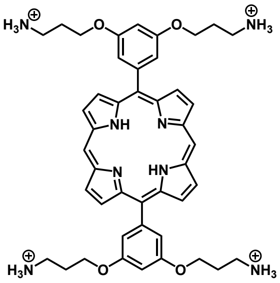

^{1}H NMR (600 MHz, CD_3OD+D_2O) δ 10.95 (s, 2H), 9.79 (d, J = 4.7 Hz, 4H), 9.32 (d, J = 4.7 Hz, 4H), 7.65 (d, J = 2.2 Hz, 4H), 7.24 (t, J = 2.2 Hz, 2H), 4.46 (t, J = 5.9 Hz, 8H), 3.37 – 3.35 (m, 8H), 2.37 – 2.35 (m, 8H).^{13}C NMR (150 MHz, CD_3OD+D_2O) δ 158.66, 146.06, 144.57, 141.93, 132.06, 130.76, 119.74, 115.82, 106.42, 101.66, 65.81, 37.39, 26.99. HRMS: m/z; $[M]^+$, calcd for $C_{44}H_{53}N_8O_4^{3+}$: 252.4725; found: 252.4725.

Compound **3C-s**

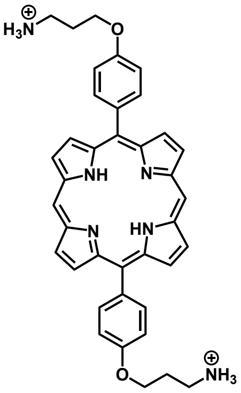

^{1}H NMR (600 MHz, CD_3OD+D_2O) δ 10.80 (s, 2H), 9.69 (d, J = 4.6 Hz, 4H), 9.17 (d, J = 4.6 Hz, 4H), 8.31 (d, J = 8.5 Hz, 4H), 7.59 (d, J = 8.5 Hz, 4H), 4.53 (t, J = 5.6 Hz, 4H), 3.42 (t, J = 7.5 Hz, 4H), 2.46 – 2.42 (m, 4H). ^{13}C NMR (150 MHz, CD_3OD+D_2O) δ 159.44, 146.73, 144.15, 137.03, 132.93, 131.77, 130.58, 119.96, 113.78, 106.00, 65.60, 37.45, 27.10. HRMS: m/z; $[M]^+$, calcd for $C_{38}H_{38}N_6O_2^{2+}$: 305.1523; found: 305.1520.

Compound **4C**

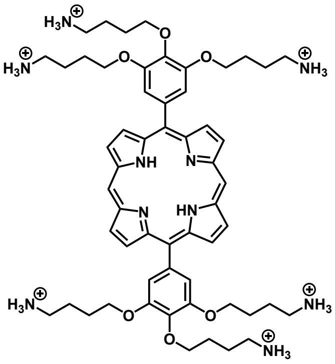

^{1}H NMR (600 MHz, CD_3OD+D_2O) *δ* 11.03 (s, 2H), 9.75 (s, 4H), 9.30 (s, 4H), 7.84 (s, 5H), 4.44 – 4.41 (m, 4H), 4.36 (t, J = 6.2 Hz, 8H), 3.22 (t, J = 6.9 Hz, 4H), 3.12 (t, J = 7.7 Hz, 8H), 2.11 – 2.04 (m, 12H), 2.02 (t, J = 5.8 Hz, 4H), 1.98 – 1.89 (m, 8H).^{13}C NMR (151 MHz, CD_3OD+D_2O) δ 151.46, 145.61, 143.08, 137.73, 136.13,

131.43, 130.51, 116.82, 73.69, 68.92, 39.41, 39.28, 26.68, 25.83, 23.93, 23.83. HRMS: m/z; $[M]^+$, calcd for $C_{56}H_{79}N_{10}O_6^{3+}$: 329.2056; found: 329.2055.

Compound **5C**

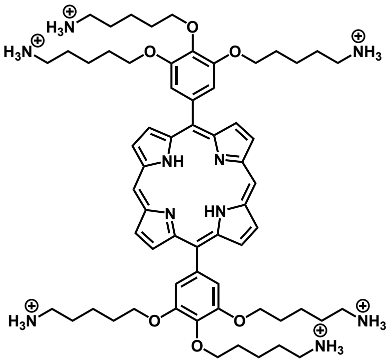

^{1}H NMR (600 MHz, CD_3OD+D_2O) δ 10.85 (s, 2H), 9.72 (s, 4H), 9.29 (s, 4H), 7.80 (s, 4H), 4.48 – 4.42 (m, 4H), 4.35 (t, J = 6.6 Hz, 8H), 3.18 (t, J = 7.8 Hz, 4H), 3.08 (t, J = 7.8 Hz, 8H), 2.06 (dq, J = 27.7, 6.9 Hz, 12H), 1.93 (p, J = 7.6 Hz, 4H), 1.89 – 1.76 (m, 12H), 1.69 (h, J = 7.9, 7.4 Hz, 8H). ^{13}C NMR (151 MHz, CD_3OD+D_2O) δ 153.11, 153.05, 139.19, 137.52, 133.47, 132.05, 121.36, 116.79, 107.93, 75.29, 70.51, 40.91, 40.80, 30.80, 29.90, 28.47, 28.23, 24.28, 24.10. HRMS: m/z; $[M]^+$, calcd for $C_{62}H_{93}N_{10}O_6^{5+}$: 214.7451; found: 214.7451.

Compound **6C**

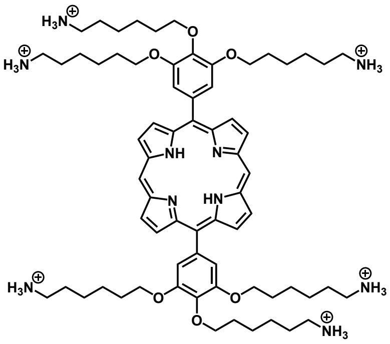

^{1}H NMR (600 MHz, CD_3OD+D_2O) δ 10.82 (s, 2H), 9.74 (d, J = 4.7 Hz, 4H), 9.26 (d, J = 4.7 Hz, 4H), 7.67 (s, 4H), 4.40 (t, J = 6.5 Hz, 4H), 4.28 (t, J = 6.2 Hz, 8H), 3.12 (t, J = 7.5 Hz, 4H), 3.00 (t, J = 7.6 Hz, 8H), 2.01 – 1.98 (m, 12H), 1.78 – 1.73 (m, 12H), 1.77 – 1.75 (m, 12H), 1.57 – 1.53 (m, 12H). ^{13}C NMR (150 MHz, CD_3OD+D_2O) δ 151.61, 137.74, 136.28, 132.07, 130.61, 119.58, 114.84, 106.10, 73.83, 69.16, 39.54, 39.39, 29.82, 28.88, 27.20, 27.04, 26.11, 25.81, 25.54, 25.32. HRMS: m/z; $[M]^+$, calcd for $C_{68}H_{105}N_{10}O_6^{5+}$: 231.5638; found: 231.5638.

Compound **5C-O**

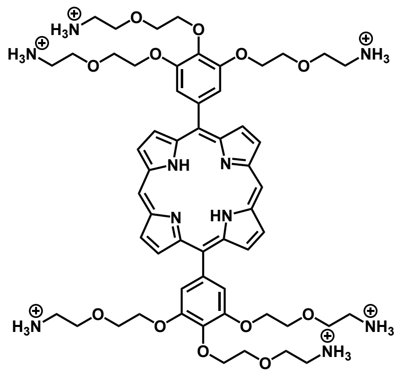

^{1}H NMR (600 MHz, CD_3OD+D_2O) δ 10.90 (s, 2H), 9.74 (s, 4H), 9.28 (d, J = 4.3 Hz, 4H), 7.81 (s, 4H), 4.64 – 4.58 (m, 4H), 4.50 (s, 8H), 4.14 (t, J = 4.5 Hz, 4H), 4.05 (dt, J = 9.9, 4.1 Hz, 12H), 3.94 – 3.88 (m, 8H), 3.37 – 3.32 (m, 4H), 3.26 – 3.20 (m, 8H). ^{13}C NMR (151 MHz, CD_3OD) δ 151.74, 146.32, 130.31, 106.80, 72.86, 70.41, 69.46, 68.87, 66.76, 66.74, 39.39, 39.33. HRMS: m/z; $[M]^+$, calcd for $C_{56}H_{80}N_{10}O_{12}^{4+}$: 271.1484; found: 271.1480.

3.The photophysical and photochemical properties of the porphyrins.

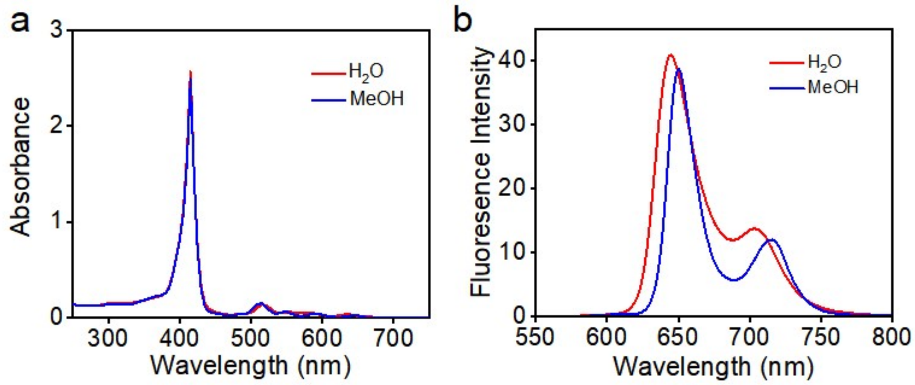

Figure S1. (a) UV−vis absorption and (b) emission spectra of **TPP 1** in different solvents. (red line: H_2O; blue line: MeOH)

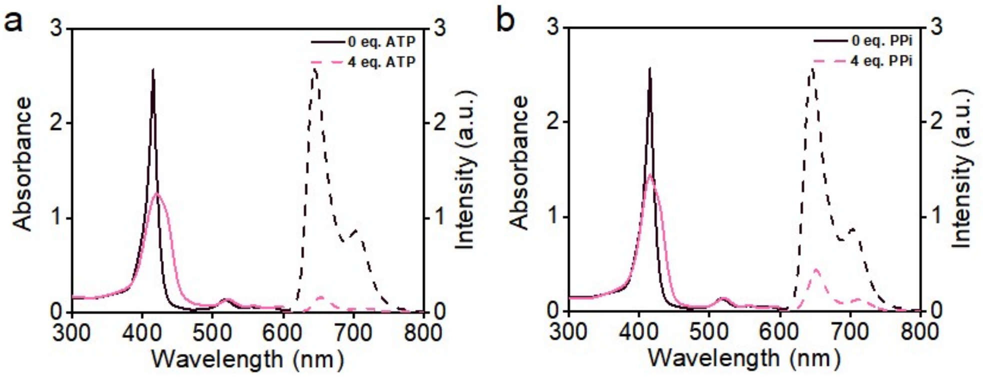

Figure S2. UV−vis absorption (solid) and emission (dash) spectra of **TPP 1** in water with increasing concentrations of (a) ATP, (b) PPi.

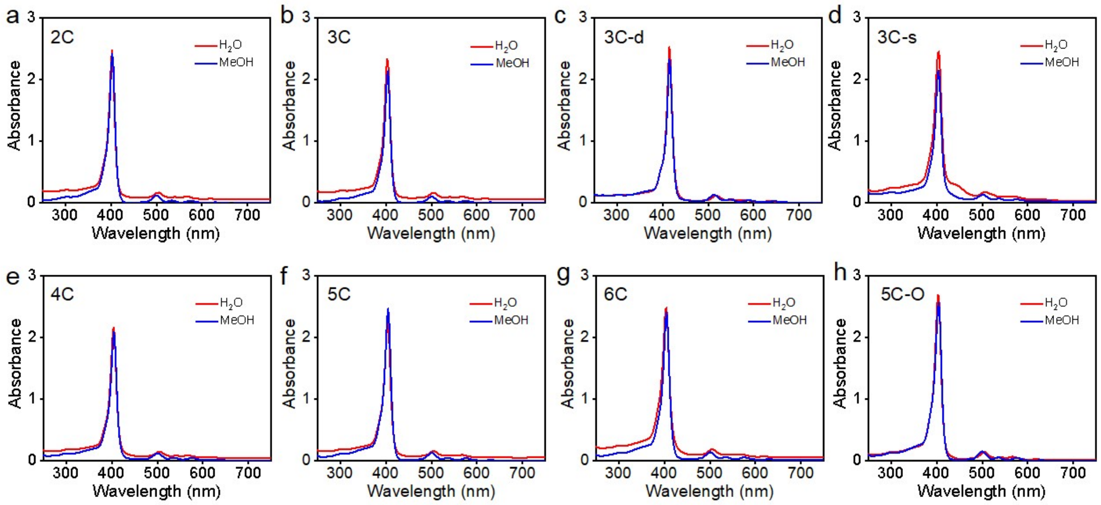

Figure S3. UV−vis absorption spectra of different porphyrins in different solvents. (red line: H_2O; blue line: MeOH)

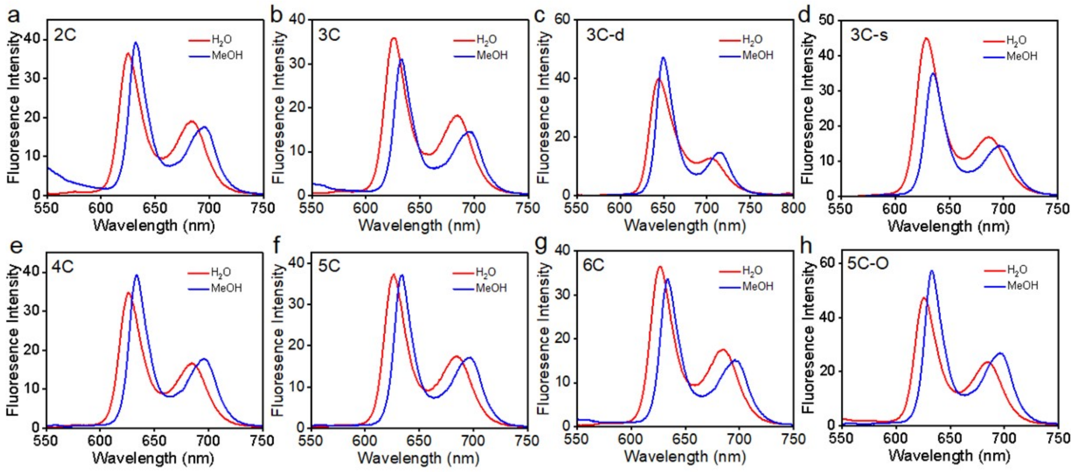

Figure S4. Emission spectra of different porphyrins in different solvents. (red line: H_2O; blue line: MeOH)

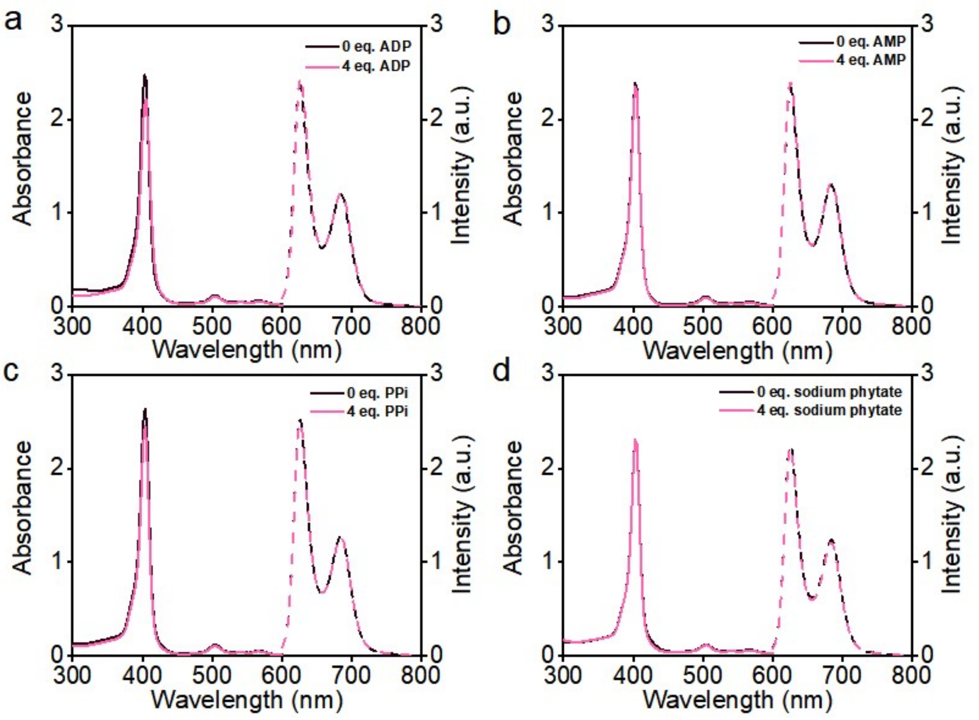

Figure S5. UV–vis absorption (solid) and emission (dash) spectra of **3C** in water with increasing concentrations of (a) ADP, (b) AMP, (c) PPi and (d) Sodium phytate.

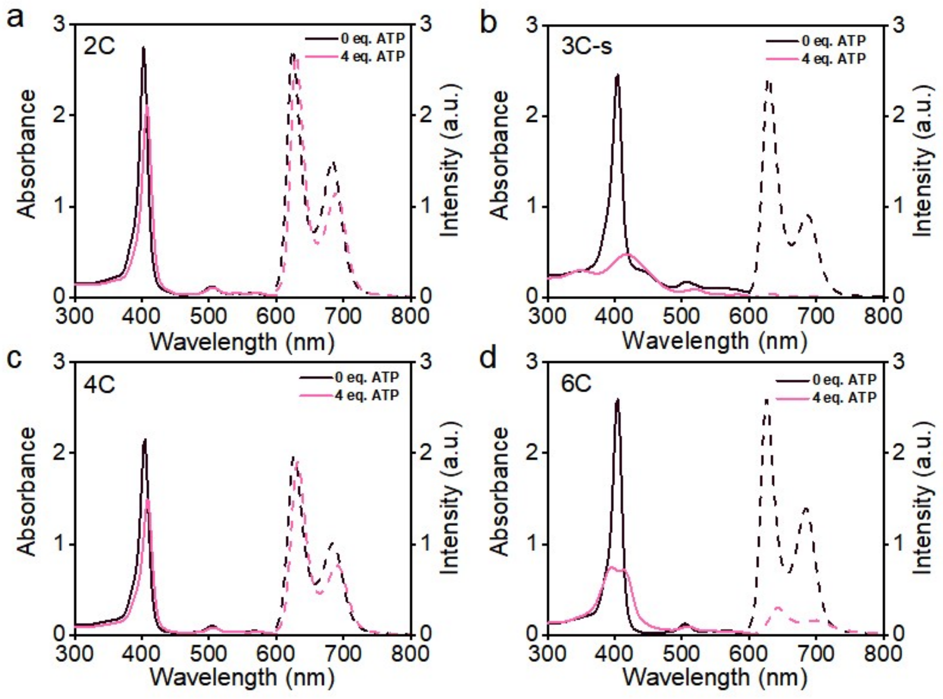

Figure S6. UV–vis absorption (solid) and emission (dash) spectra of (a) **2C**, (b) **3C-s**, (c) **4C** and (d) **6C** in water with increasing concentrations of ATP.

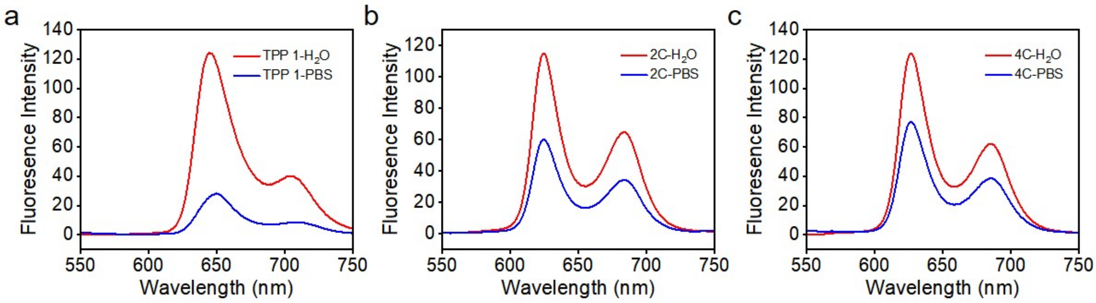

Figure S7. Fluorescence spectra of (a) **TPP 1**, (b) **2C**, (c) **4C** in H_2O or PBS.

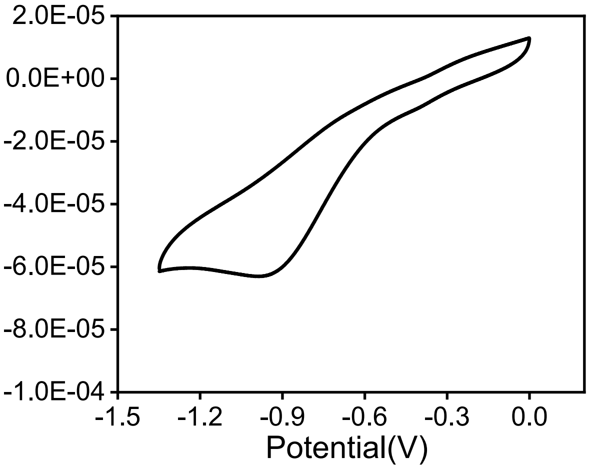

Figure S8. Cyclic voltammograms of **3C**.

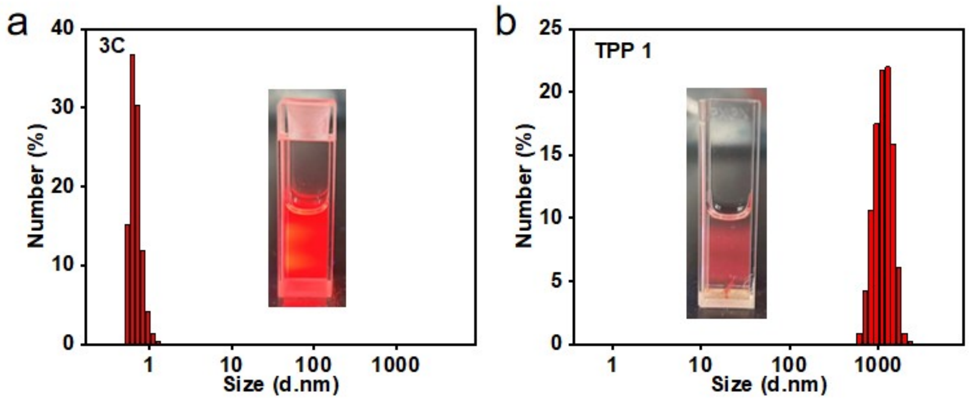

Figure S9. Size distribution of (a) **3C** and (b) **TPP 1** in PBS measured by DLS. ([3C] = [TPP 1] = 10 μM, Insets are the photographs of PSs in PBS under light irradiation)

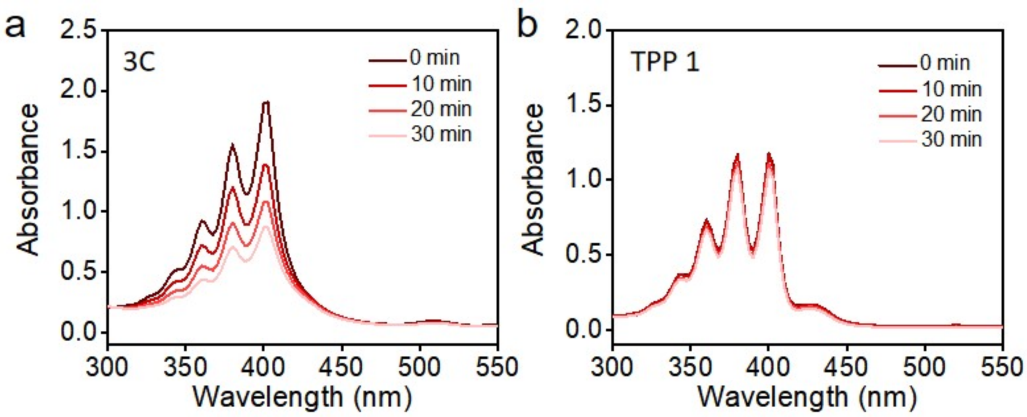

Figure S10. The absorbance of ABDA in PBS containing (a) **3C** or (b) **TPP 1** with increasing illumination time ([3C] = [TPP 1] = 10 μM, 630 nm, 50 mW/cm^2)

4. Experimental data *in vivo*

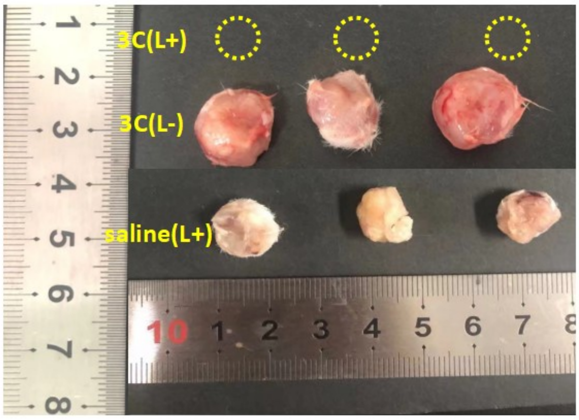

Figure S11. Images of the dissected tumors of mice from different groups after treatments.

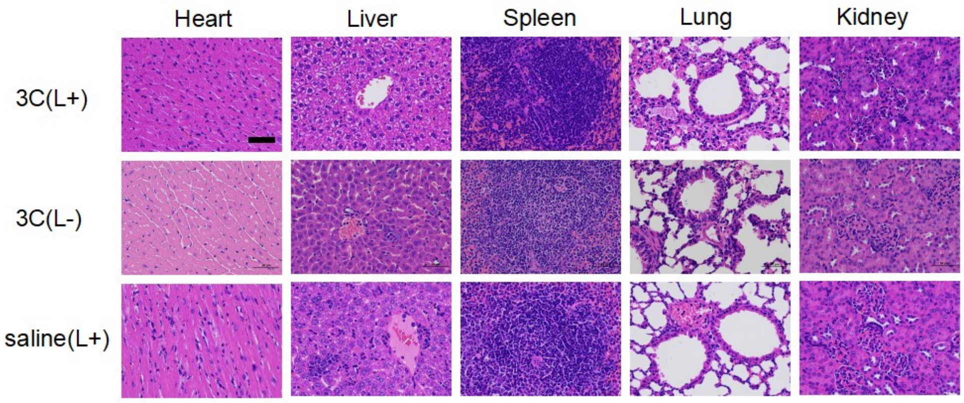

Figure S12. Hematoxylin and eosin (H&E)-stained histological section of heart, spleen, kidney, liver, lung, and tumor tissues obtained from mice of the control and PDT groups. Scale bar: 50 μm.

5. NMR spectra and HRMS

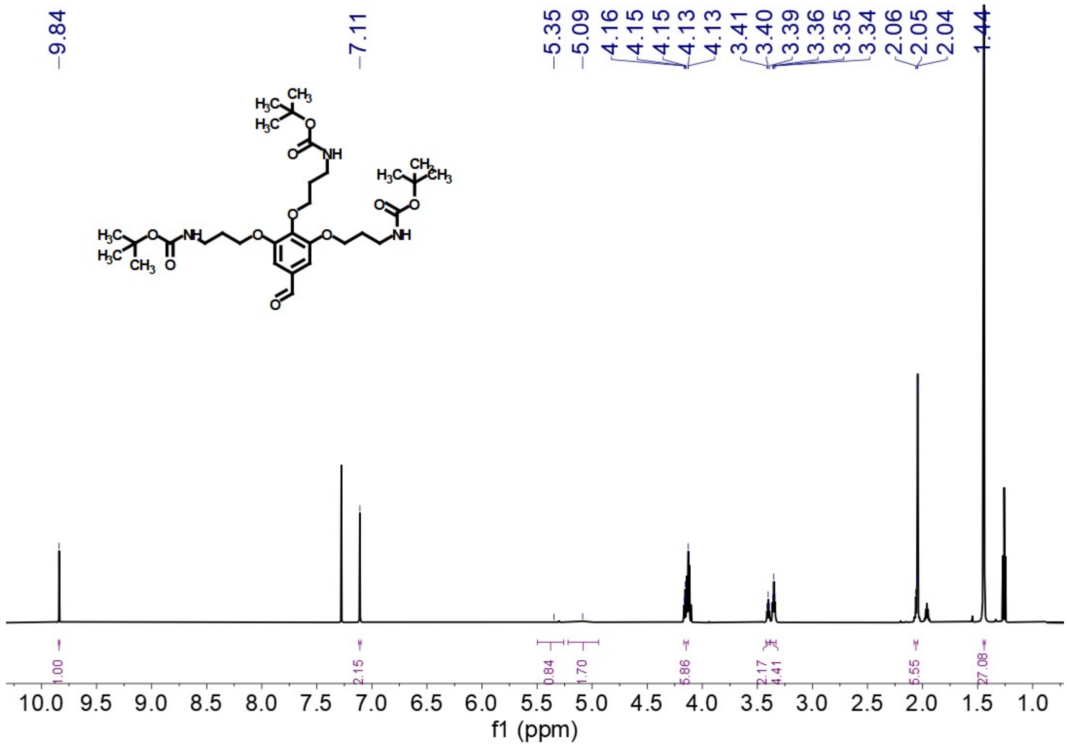

Figure S13. ^{1}H-NMR spectrum of $\mathbf{b_2}$ in $CDCl_3$.

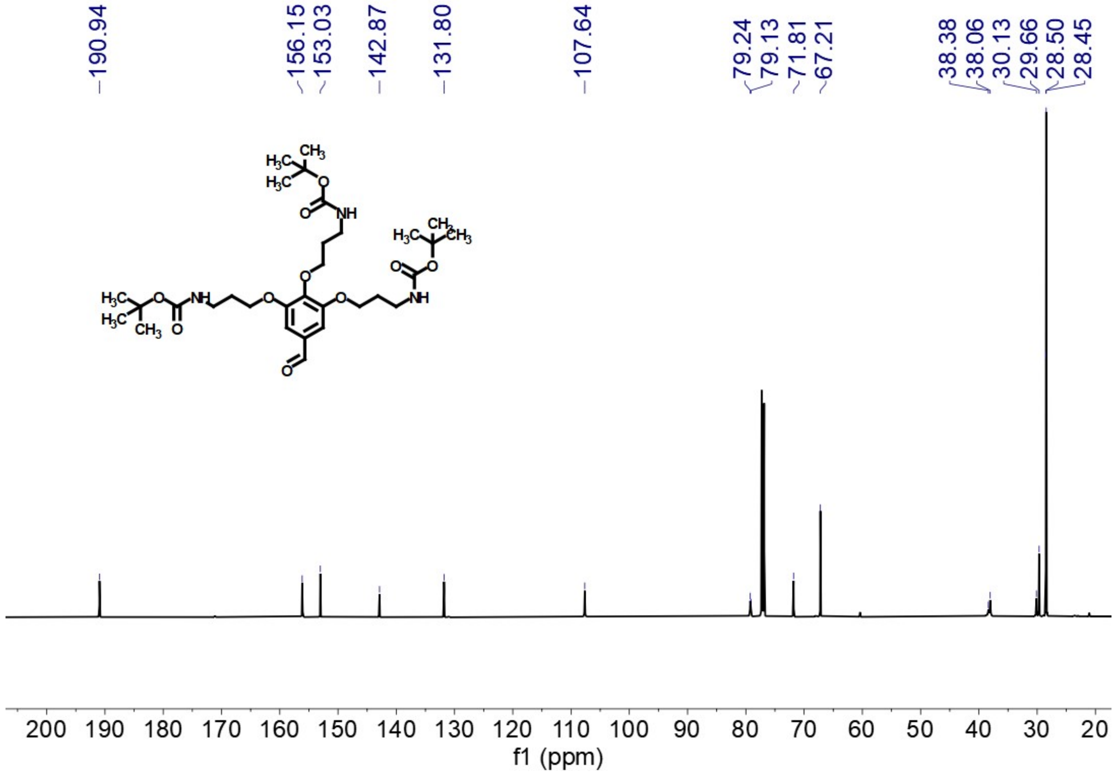

Figure S14. ^{13}C-NMR spectrum of $\mathbf{b_2}$ in $CDCl_3$.

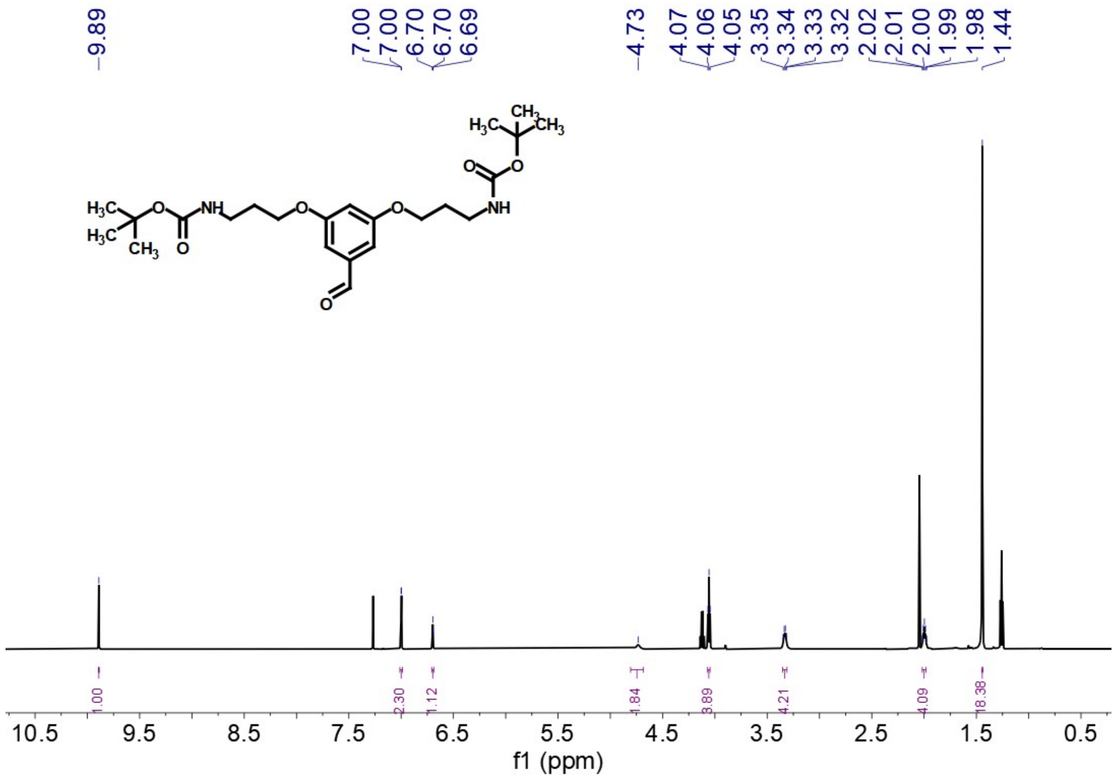

Figure S15. ^{1}H-NMR spectrum of **b$_3$** in $CDCl_3$.

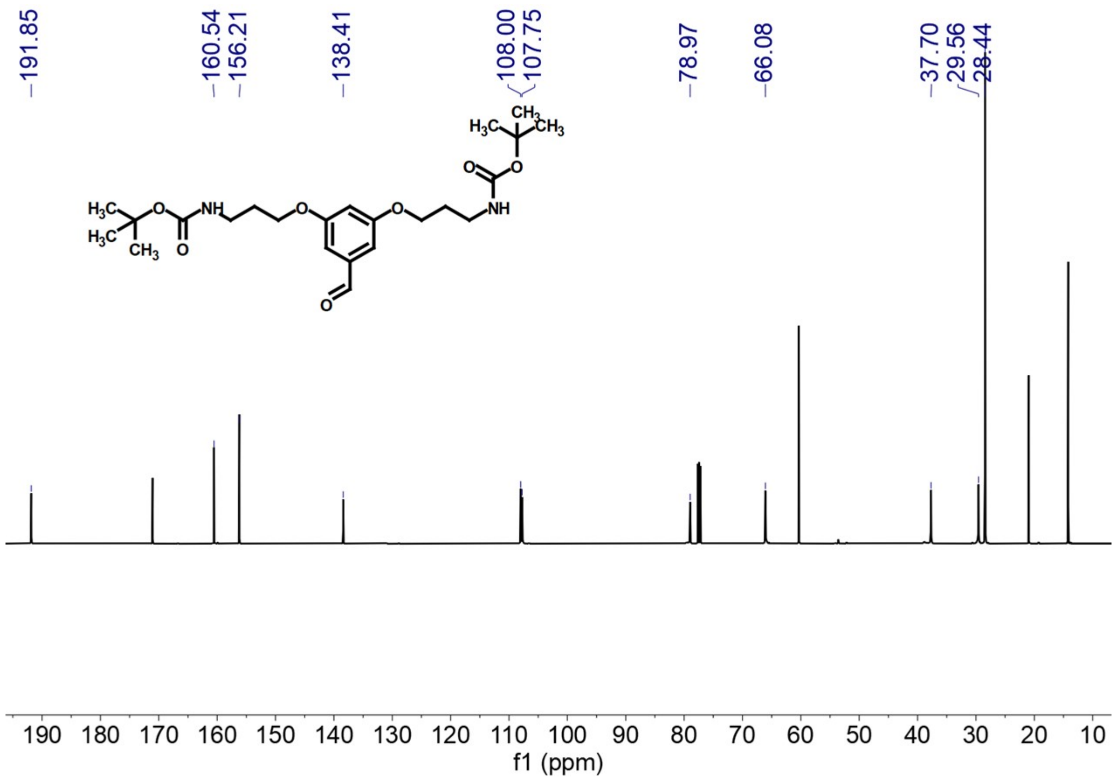

Figure S16. ^{13}C-NMR spectrum of **b$_3$** in $CDCl_3$.

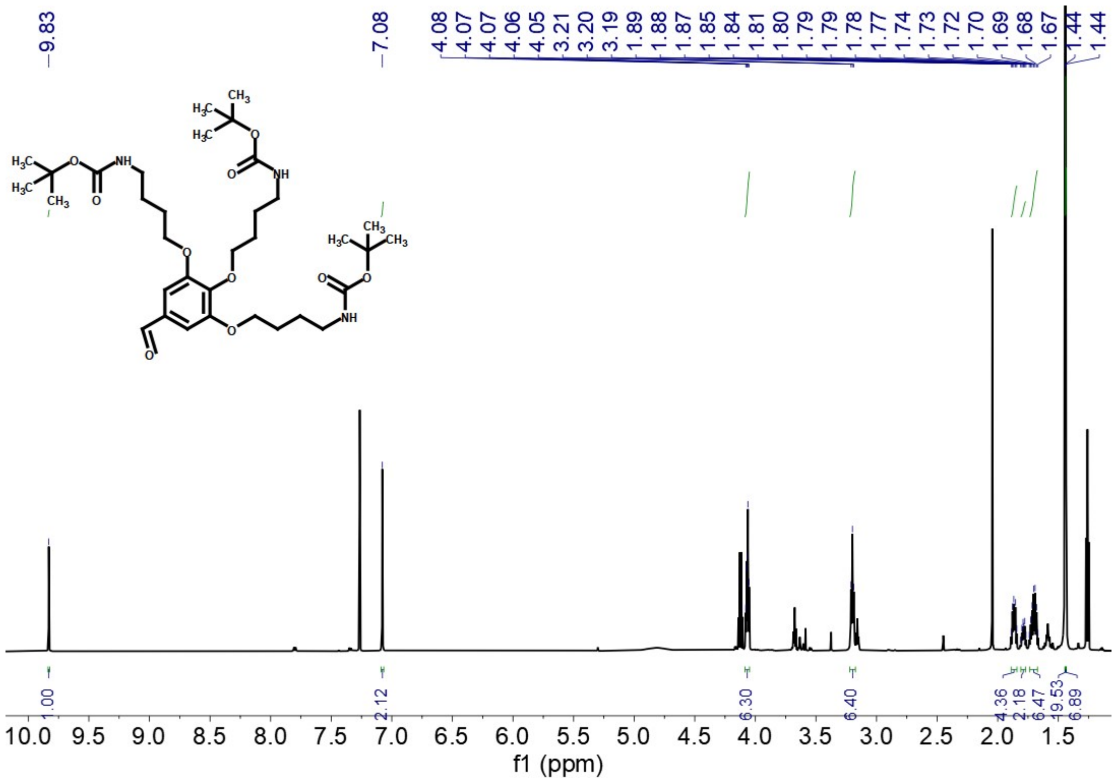

Figure S17. ^{1}H-NMR spectrum of $\mathbf{b_5}$ in $CDCl_3$.

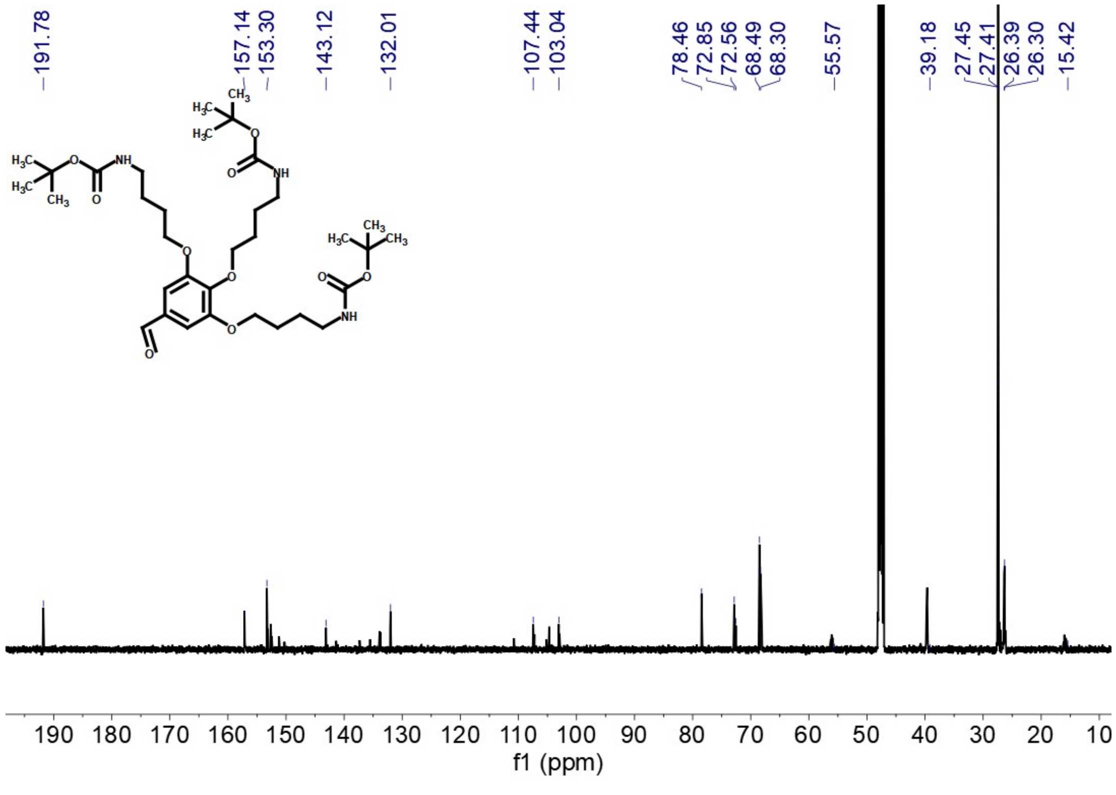

Figure S18. ^{13}C-NMR spectrum of $\mathbf{b_5}$ in $CDCl_3$.

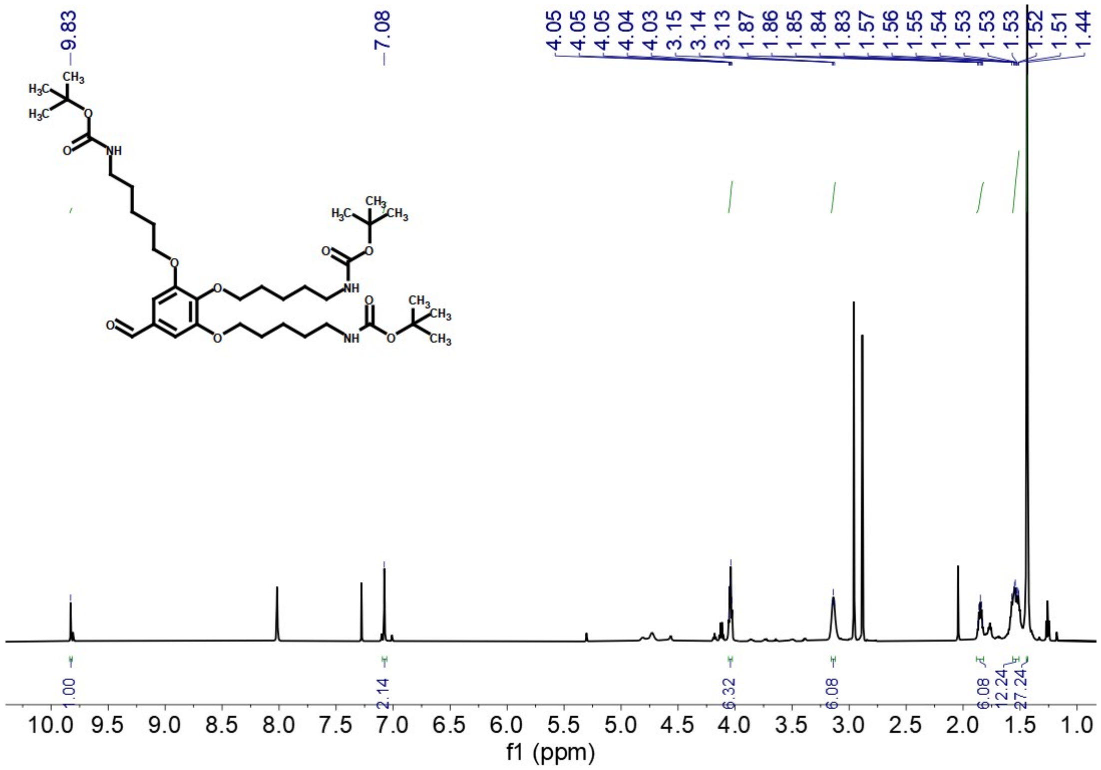

Figure S19. ^{1}H-NMR spectrum of $\mathbf{b_6}$ in $CDCl_3$.

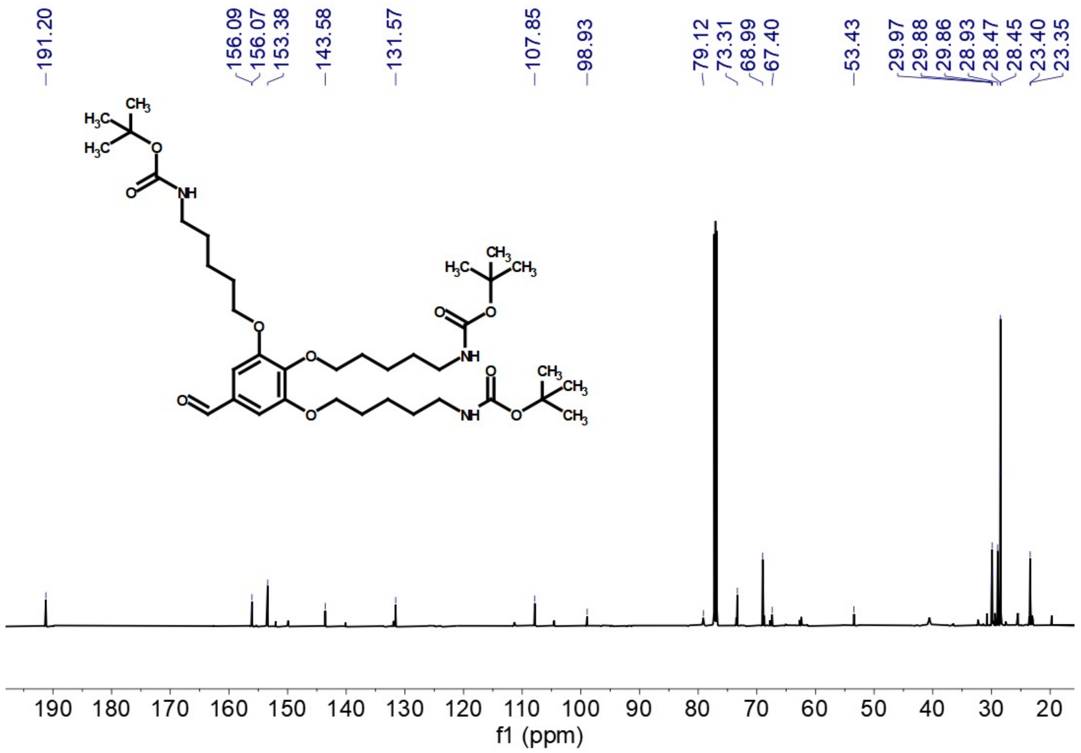

Figure S20. ^{13}C-NMR spectrum of $\mathbf{b_6}$ in $CDCl_3$.

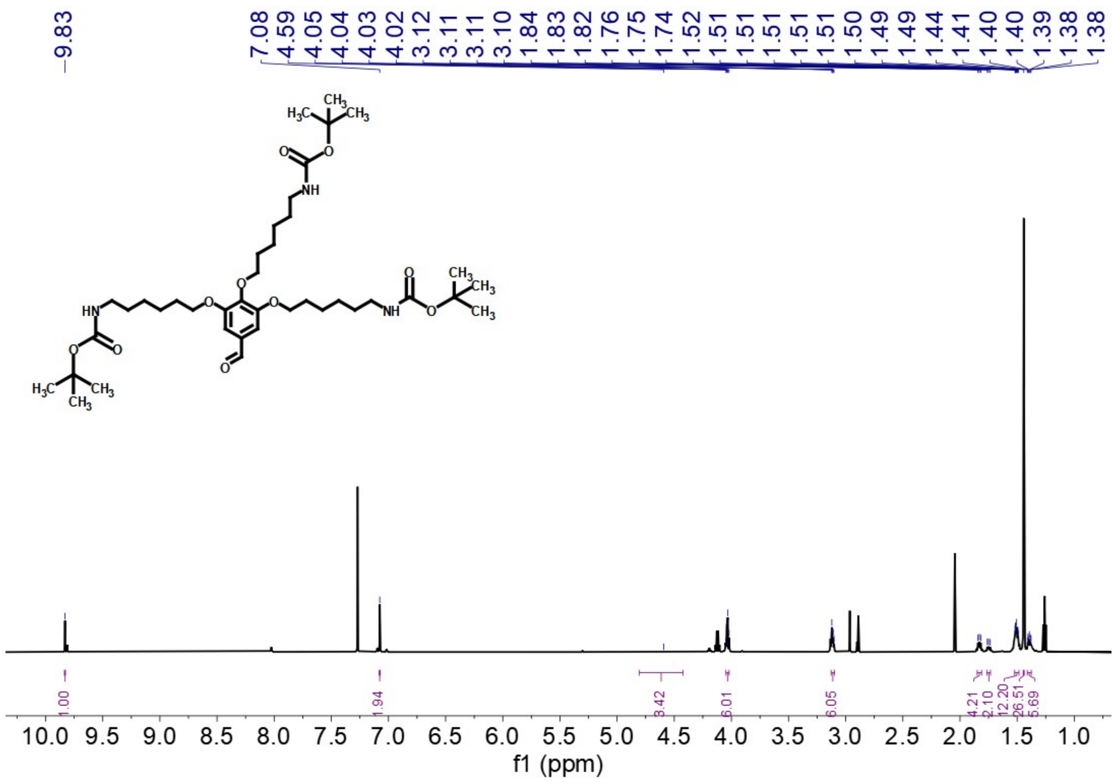

Figure S21. ^{1}H-NMR spectrum of $\mathbf{b_7}$ in $CDCl_3$.

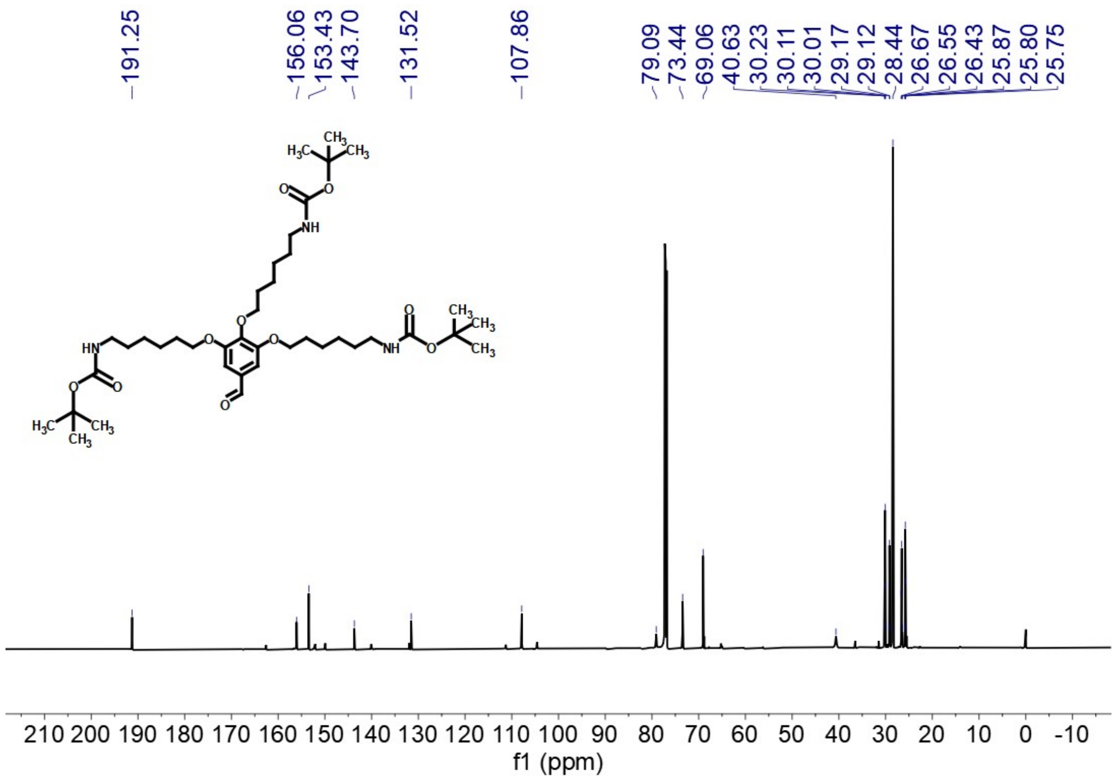

Figure S22. ^{13}C-NMR spectrum of $\mathbf{b_7}$ in $CDCl_3$.

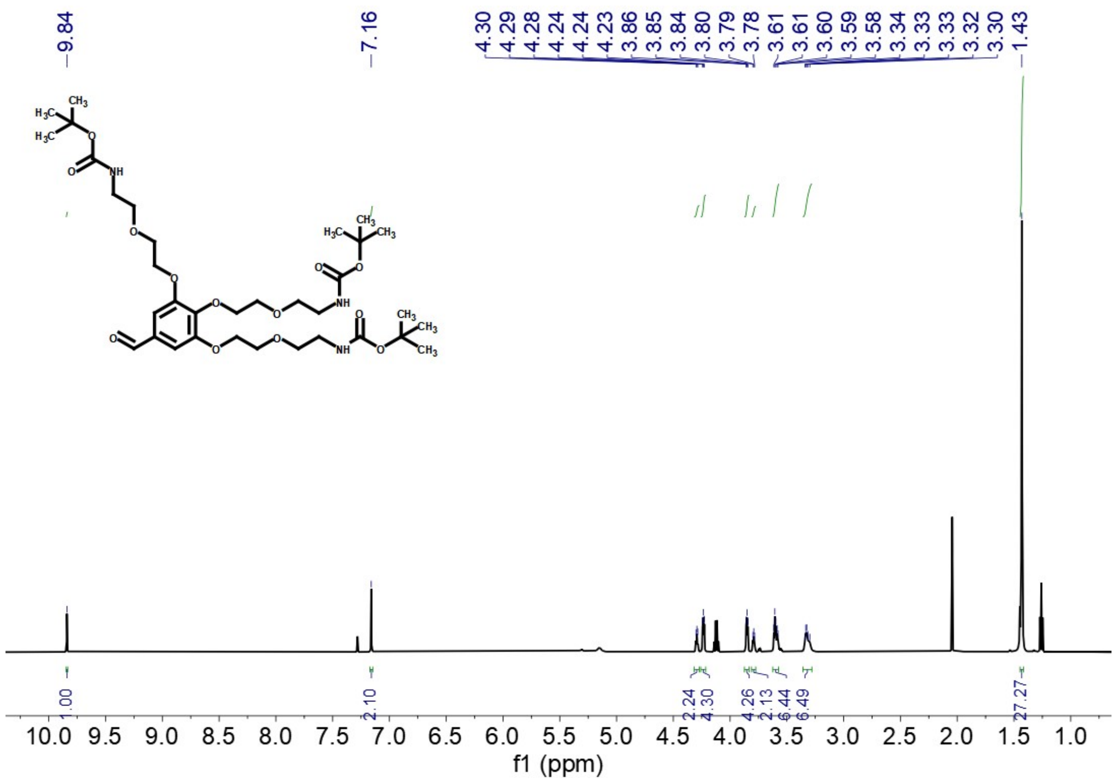

Figure S23. ^{1}H-NMR spectrum of $\mathbf{b_8}$ in $CDCl_3$.

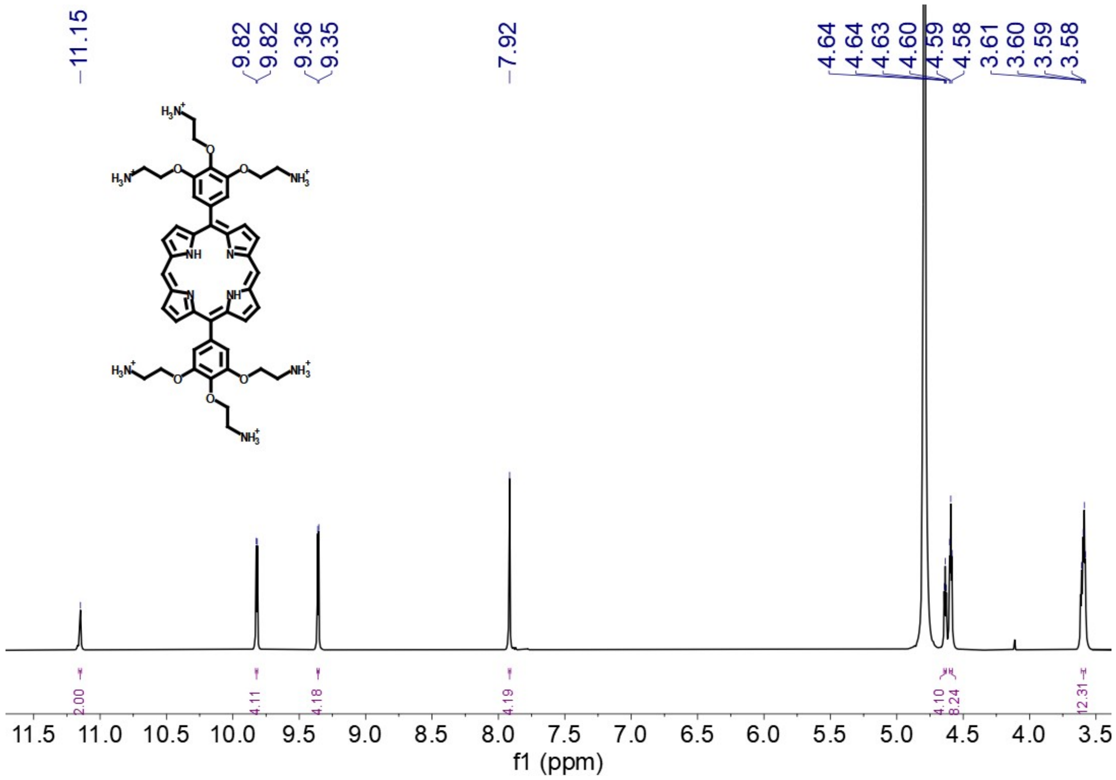

Figure S24. ^{1}H-NMR spectrum of **2C** in CD_3OD+D_2O.

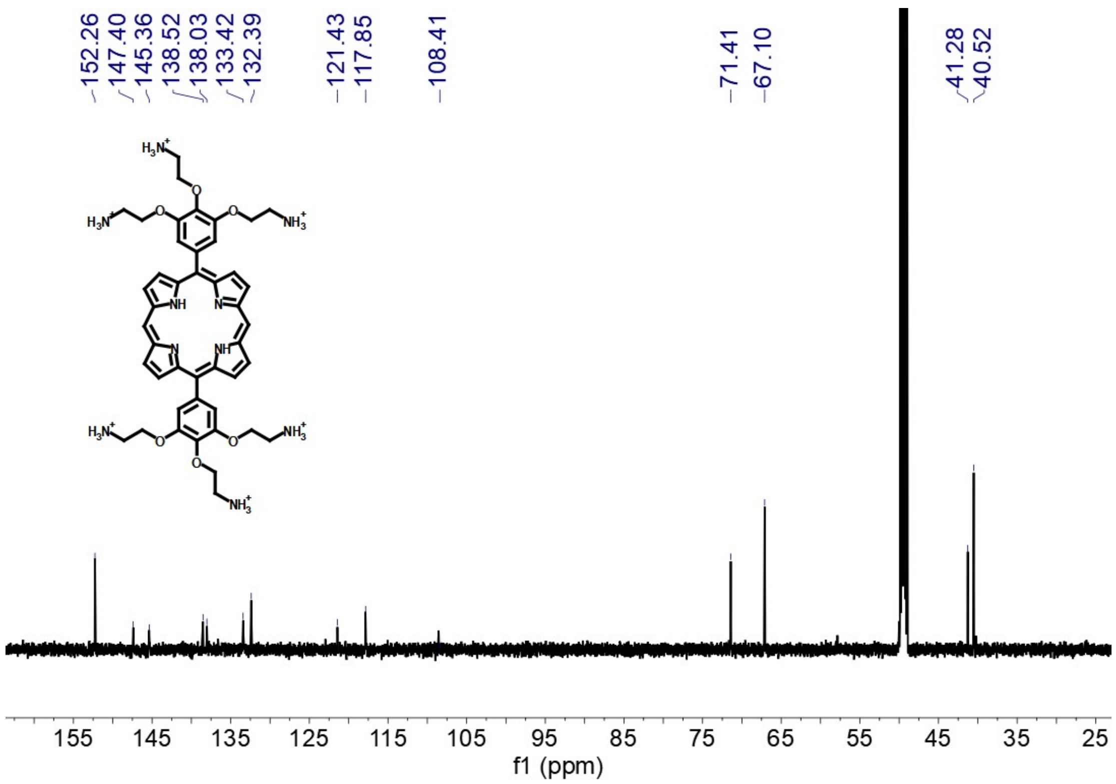

Figure S25. ^{13}C-NMR spectrum of **2C** in CD_3OD+D_2O.

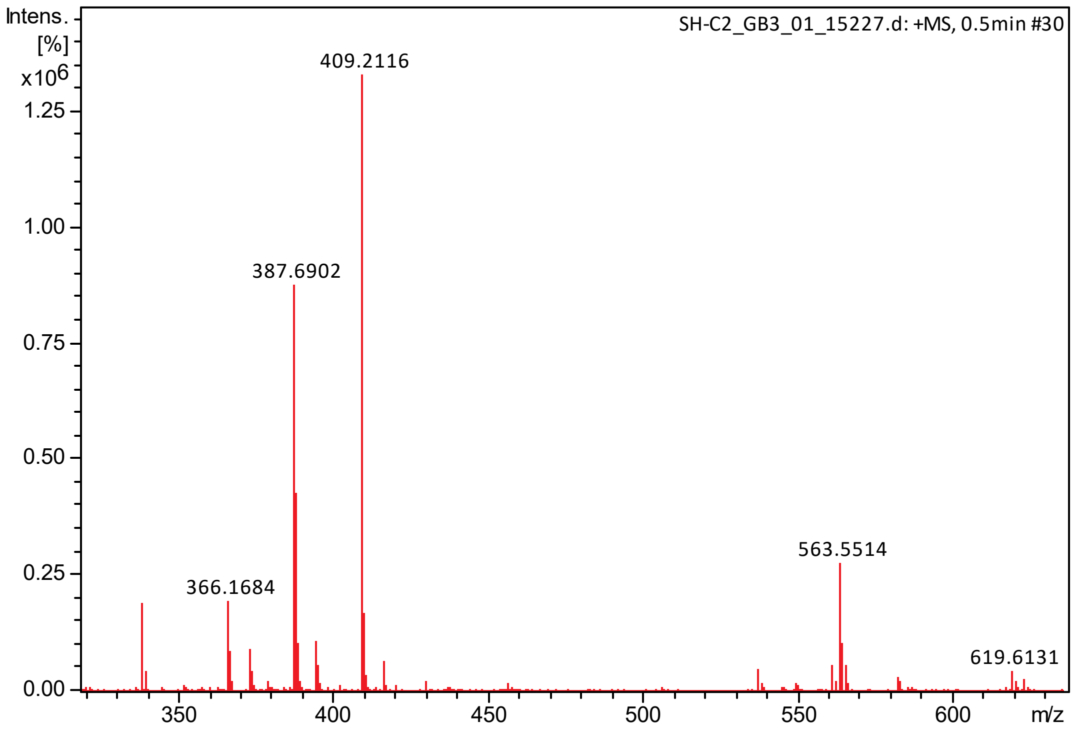

Figure S26. HRMS spectrum of **2C**.

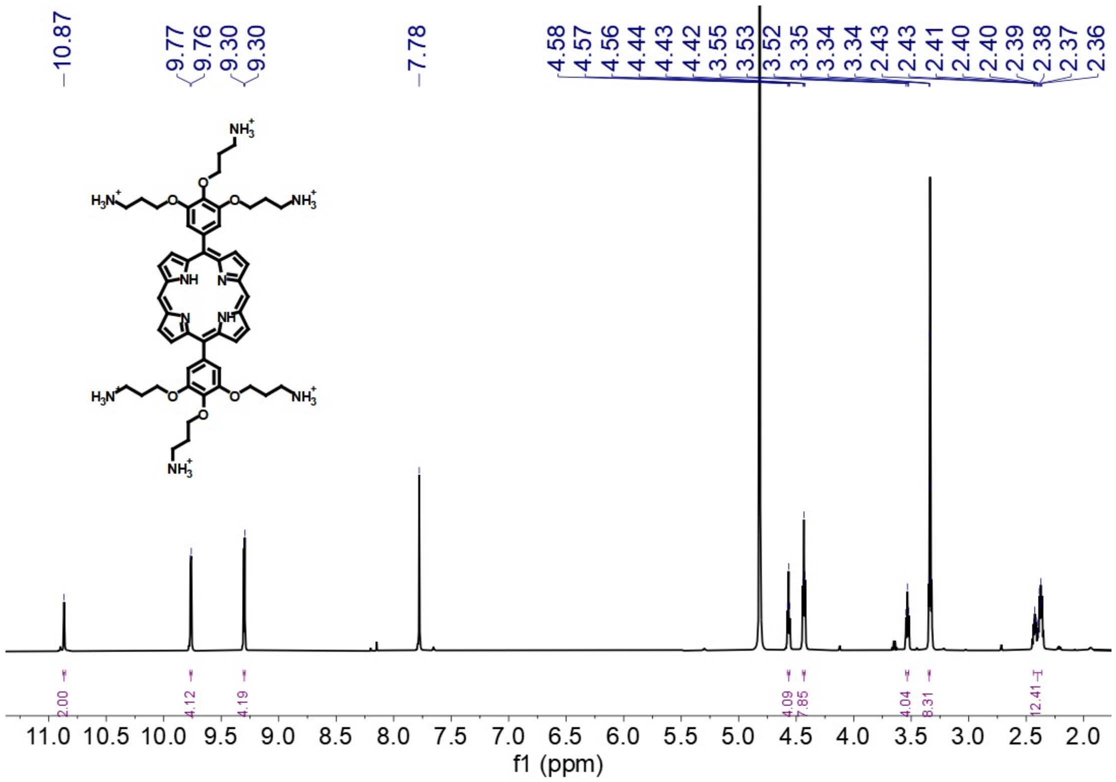

Figure S27. ^{1}H-NMR spectrum of **3C** in CD_3OD+D_2O.

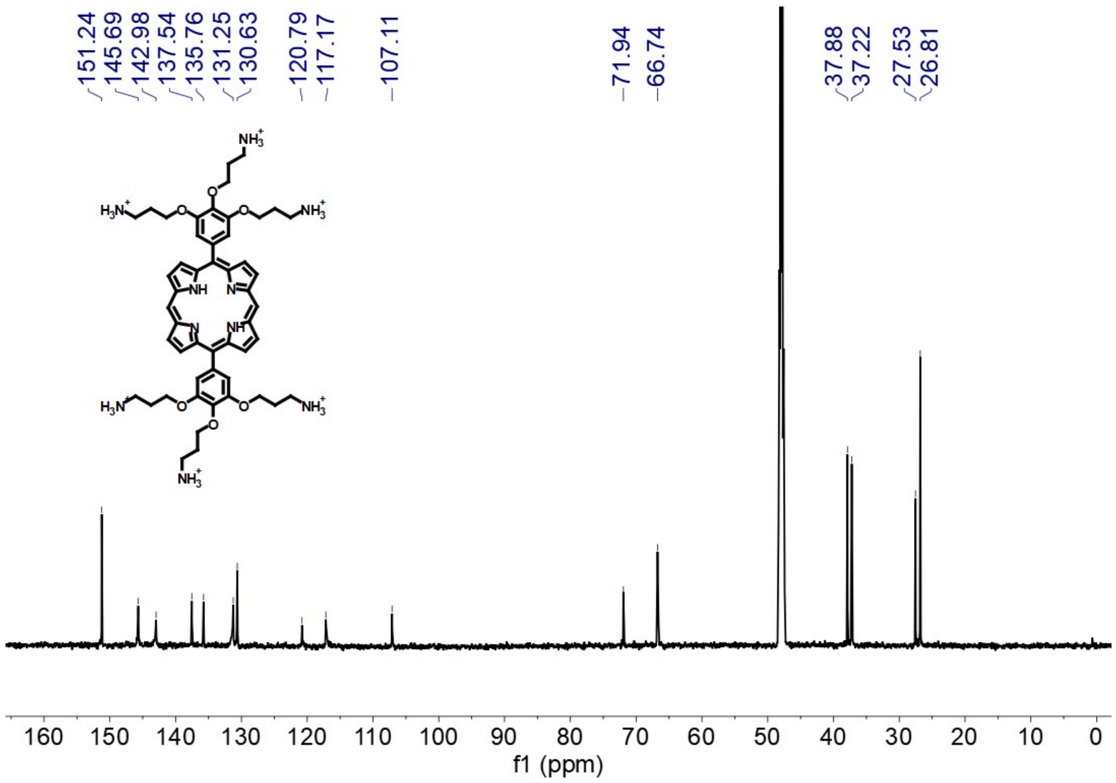

Figure S28. ^{13}C-NMR spectrum of **3C** in CD_3OD+D_2O.

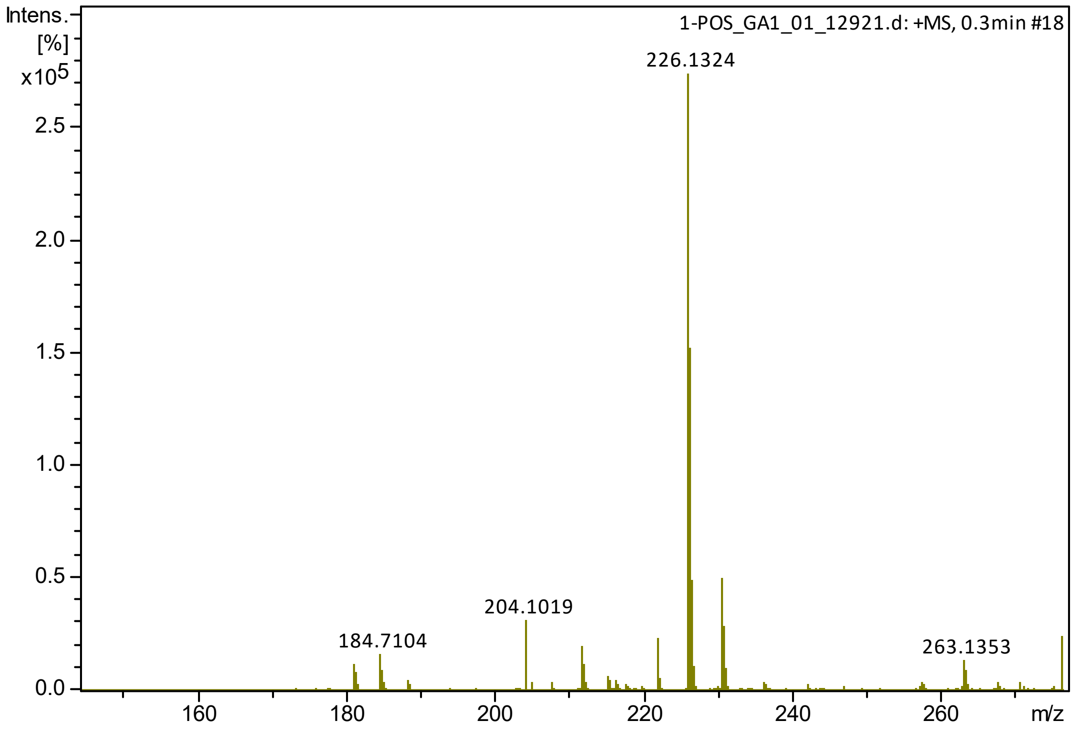

Figure S29. HRMS spectrum of **3C.**

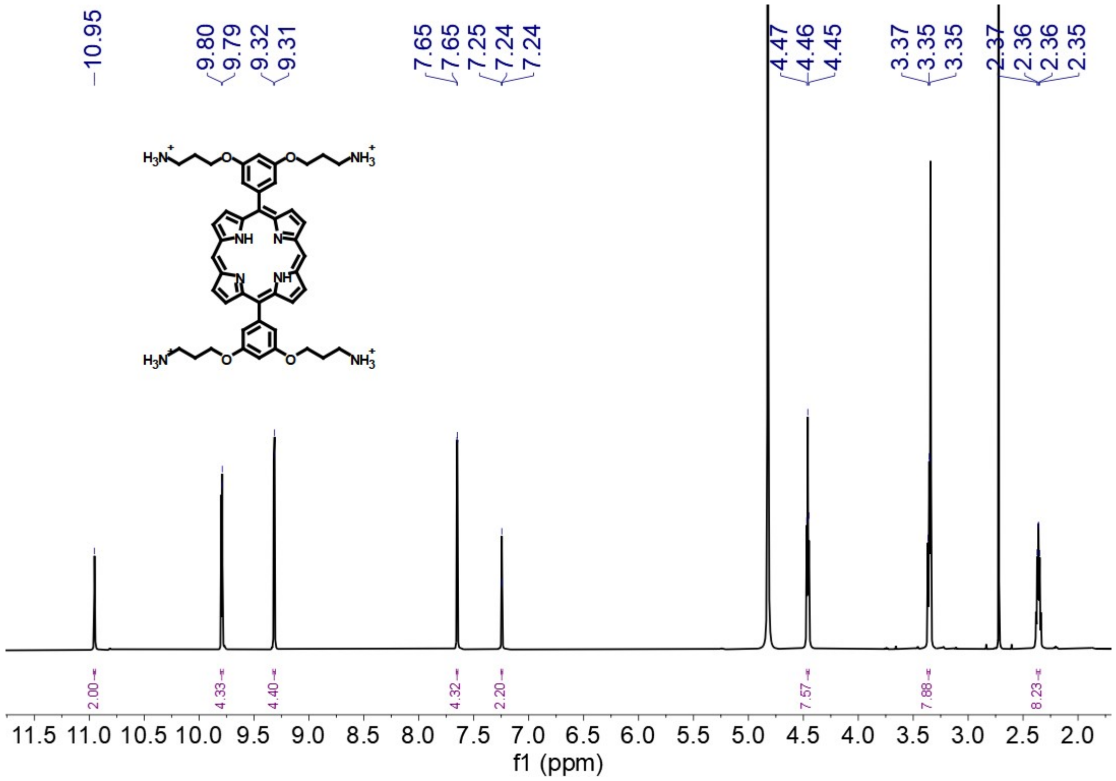

Figure S30. ^{1}H-NMR spectrum of **3C-d** in CD_3OD+D_2O.

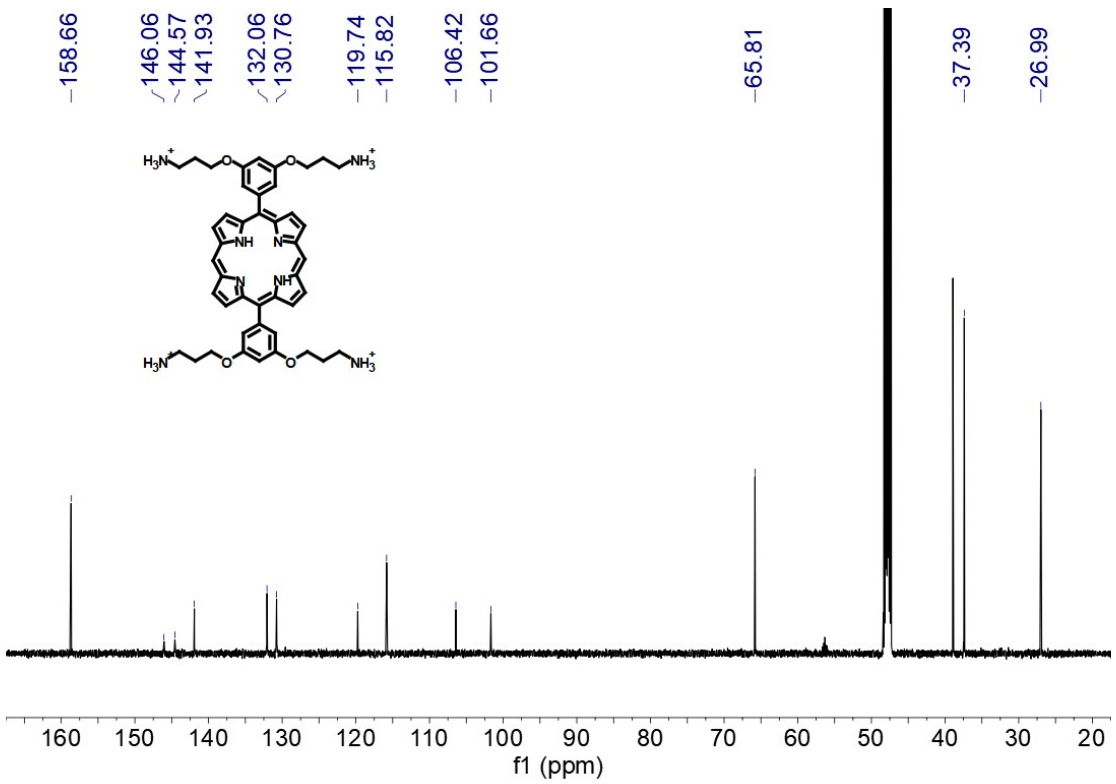

Figure S31. ^{13}C-NMR spectrum of **3C-d** in CD_3OD+D_2O.

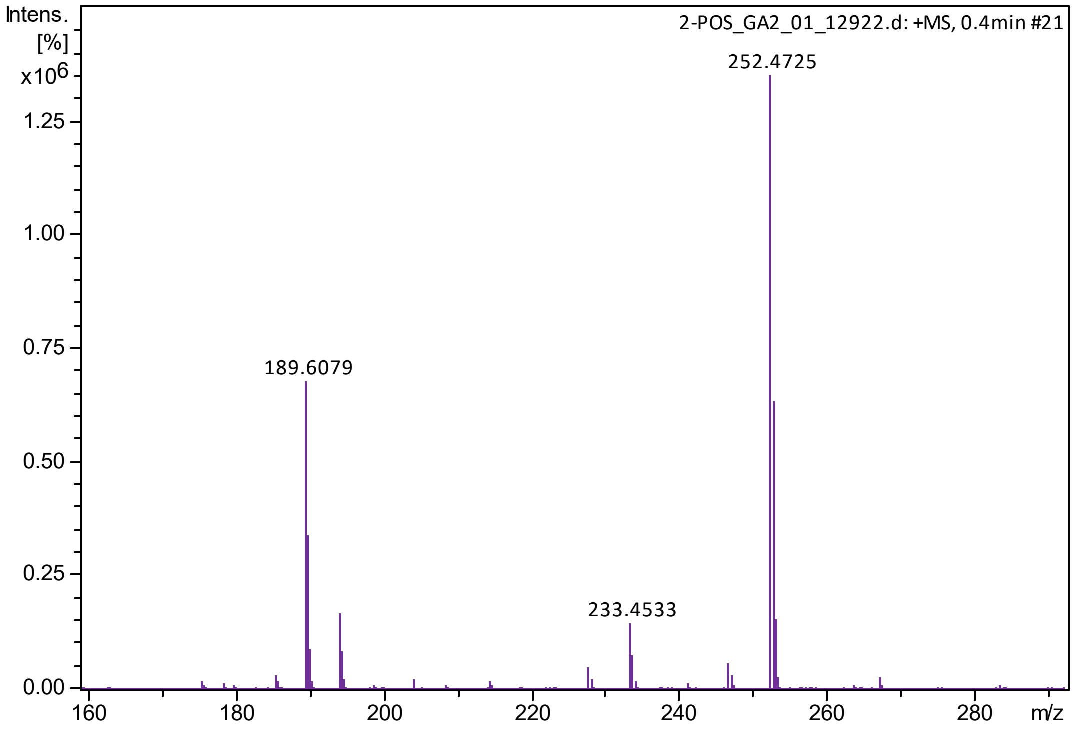

Figure S32. HRMS spectrum of **3C-d.**

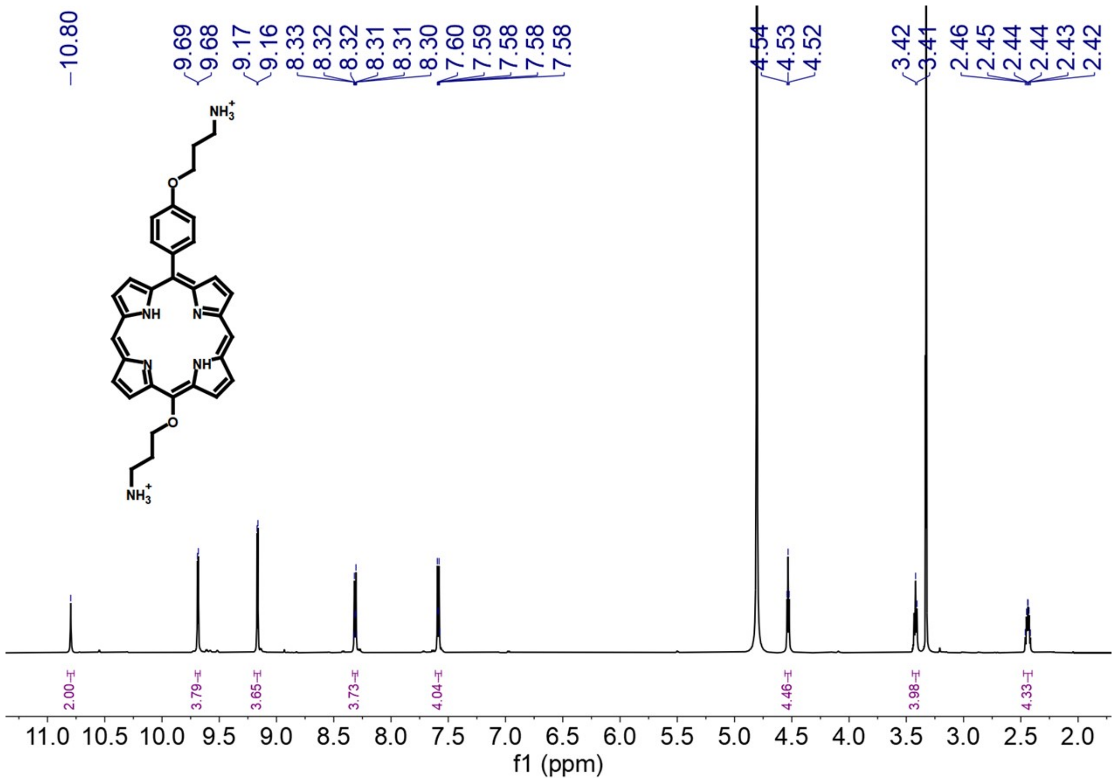

Figure S33. ^{1}H-NMR spectrum of **3C-s** in CD_3OD+D_2O.

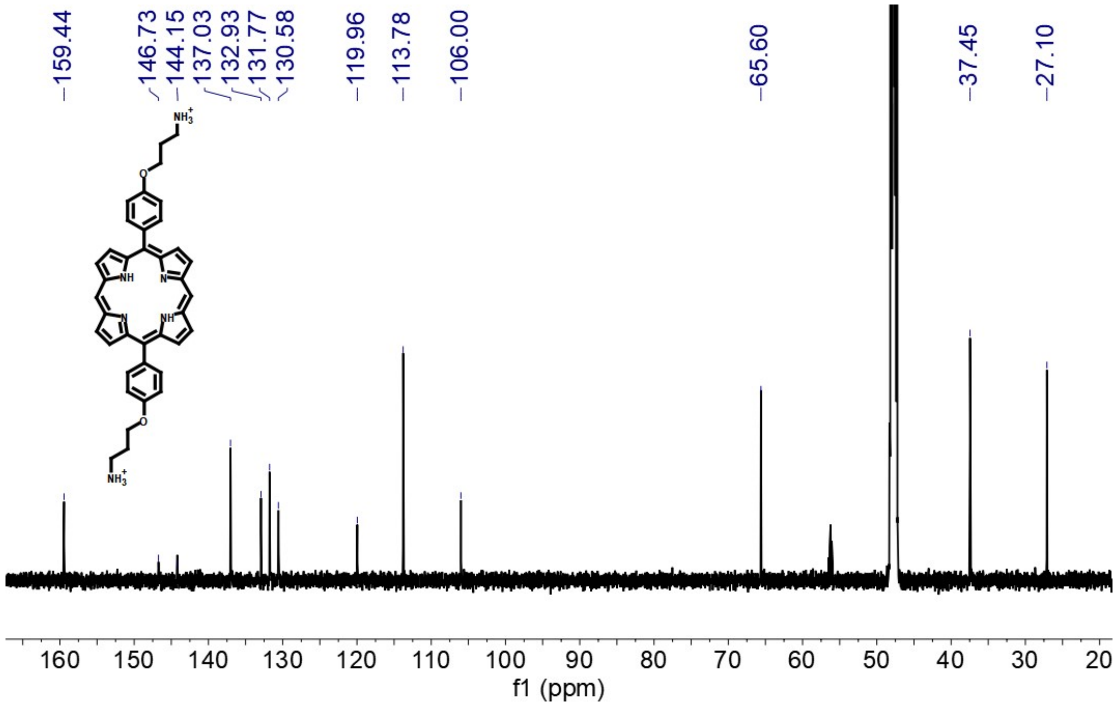

Figure S34. ^{13}C-NMR spectrum of **3C-s** in CD_3OD+D_2O.

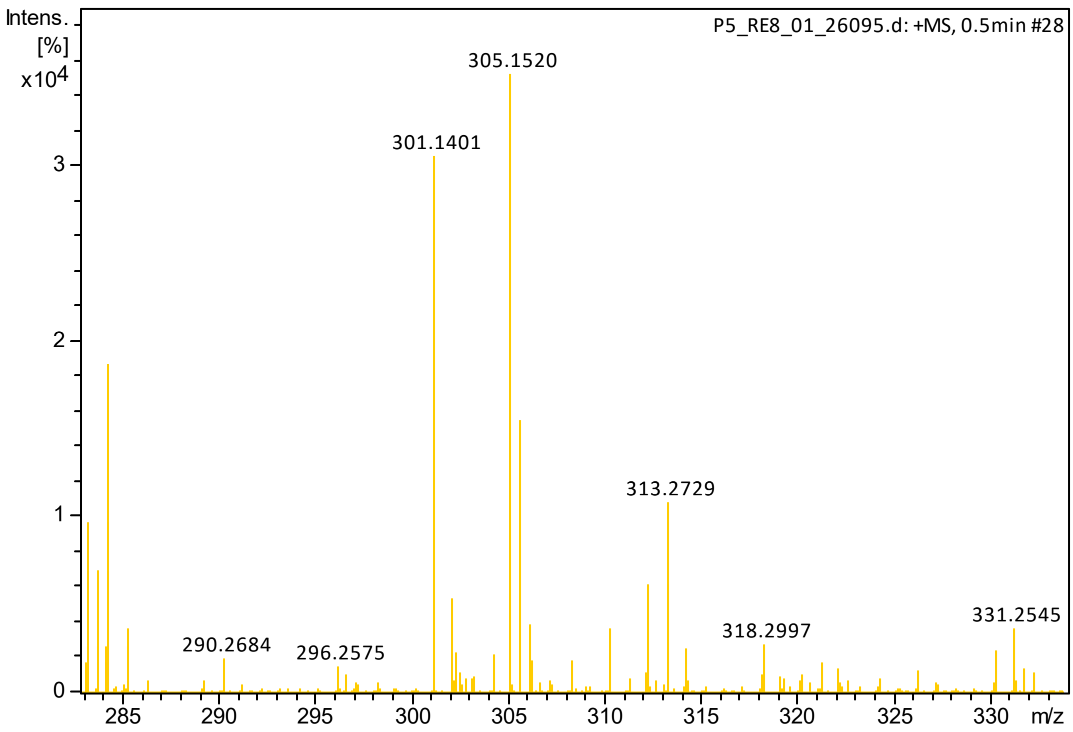

Figure S35. HRMS spectrum of **3C-s.**

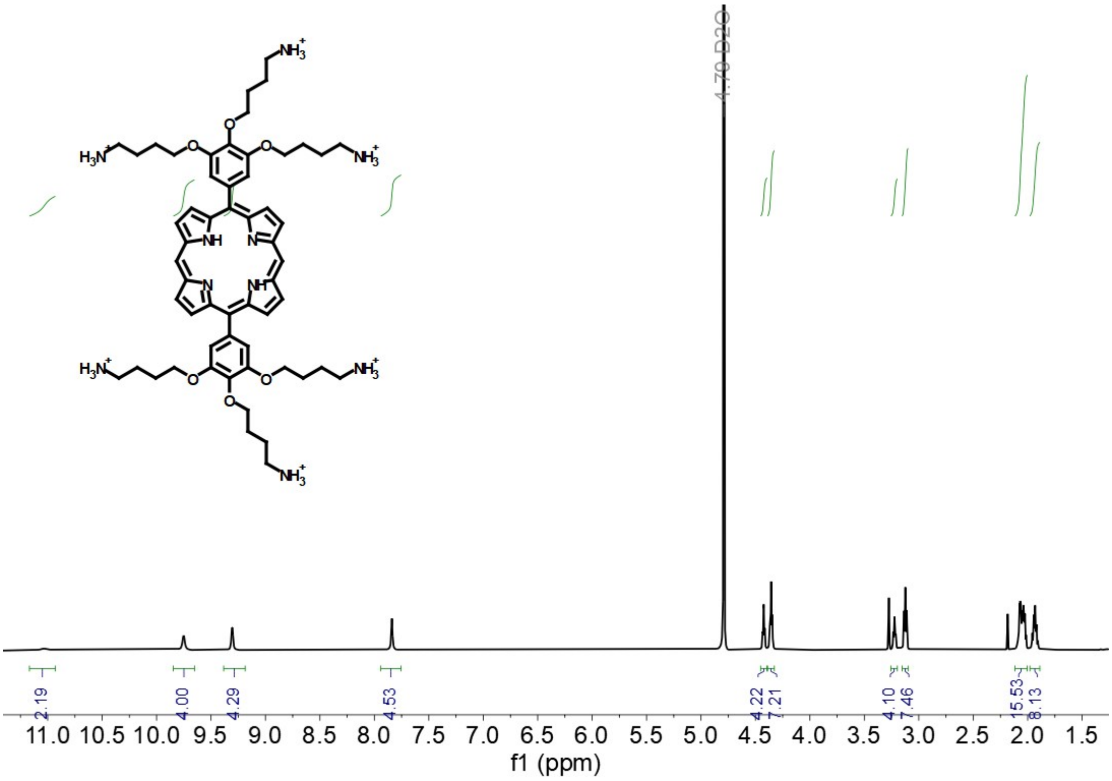

Figure S36. ^{1}H-NMR spectrum of **4C** in CD_3OD+D_2O.

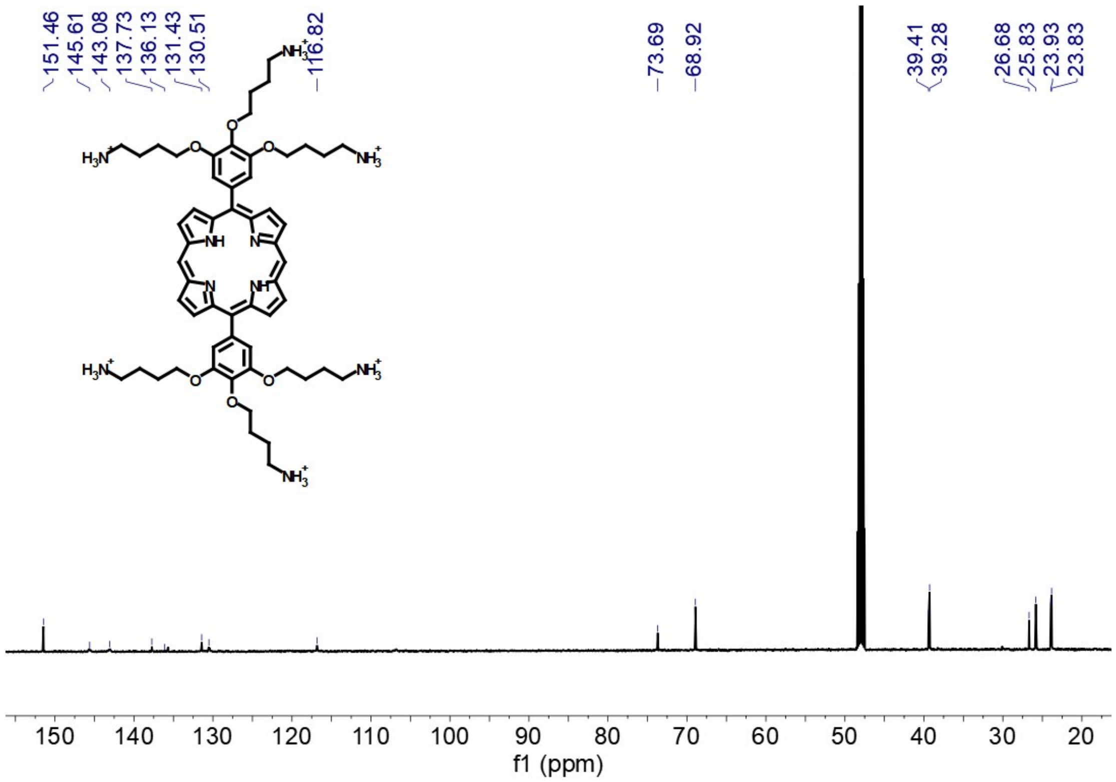

Figure S37. ^{13}C-NMR spectrum of **4C** in CD_3OD+D_2O.

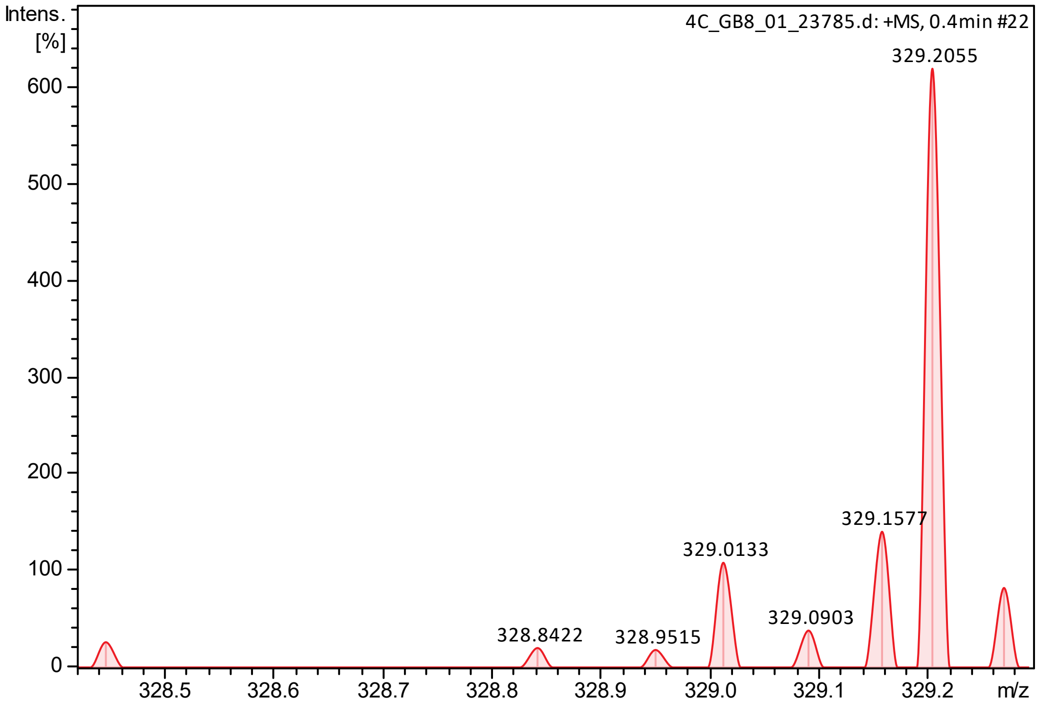

Figure S38. HRMS spectrum of **4C.**

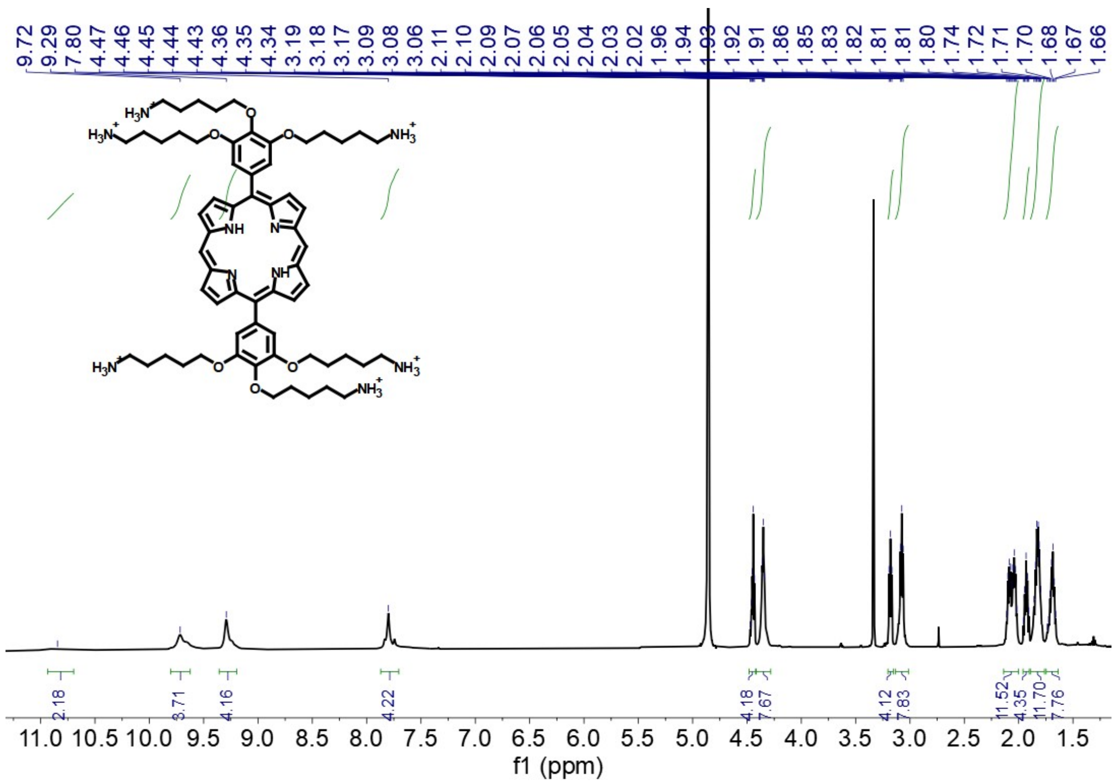

Figure S39. ^{1}H-NMR spectrum of **5C** in CD_3OD+D_2O.

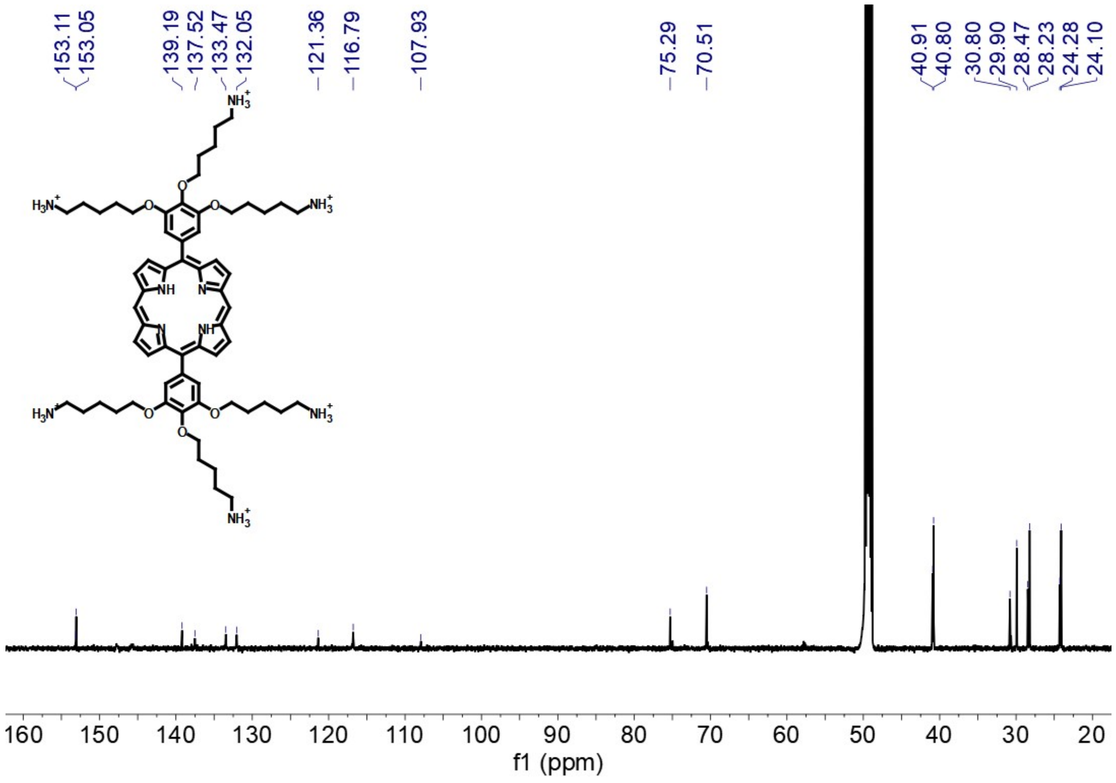

Figure S40. ^{13}C-NMR spectrum of **5C** in CD_3OD+D_2O.

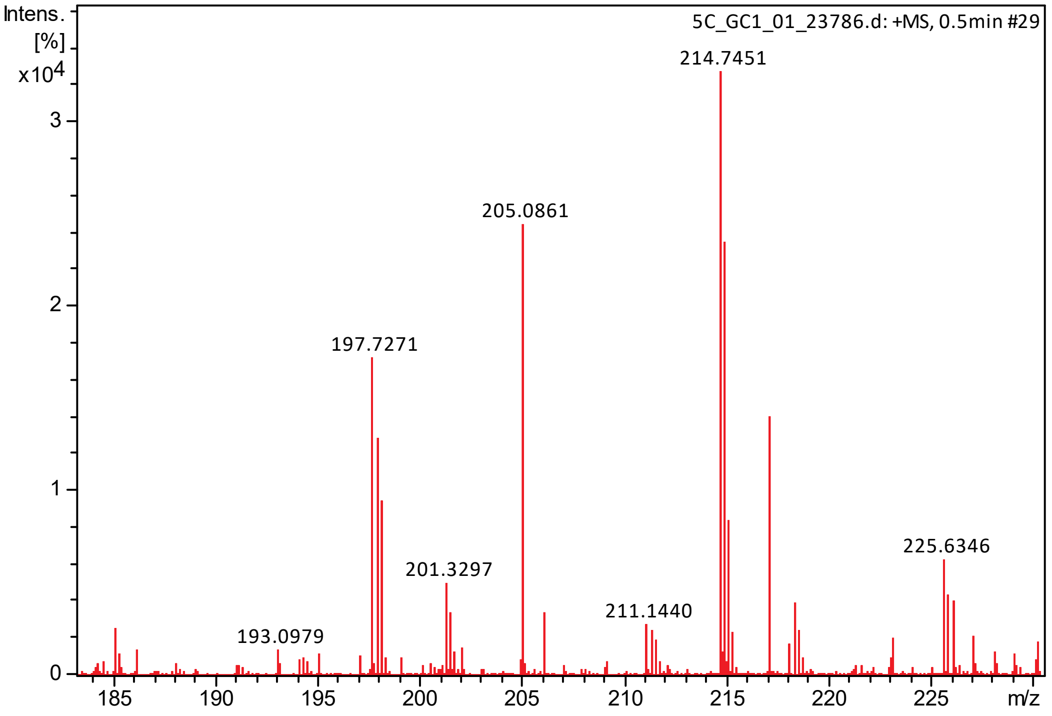

Figure S41. HRMS spectrum of **5C.**

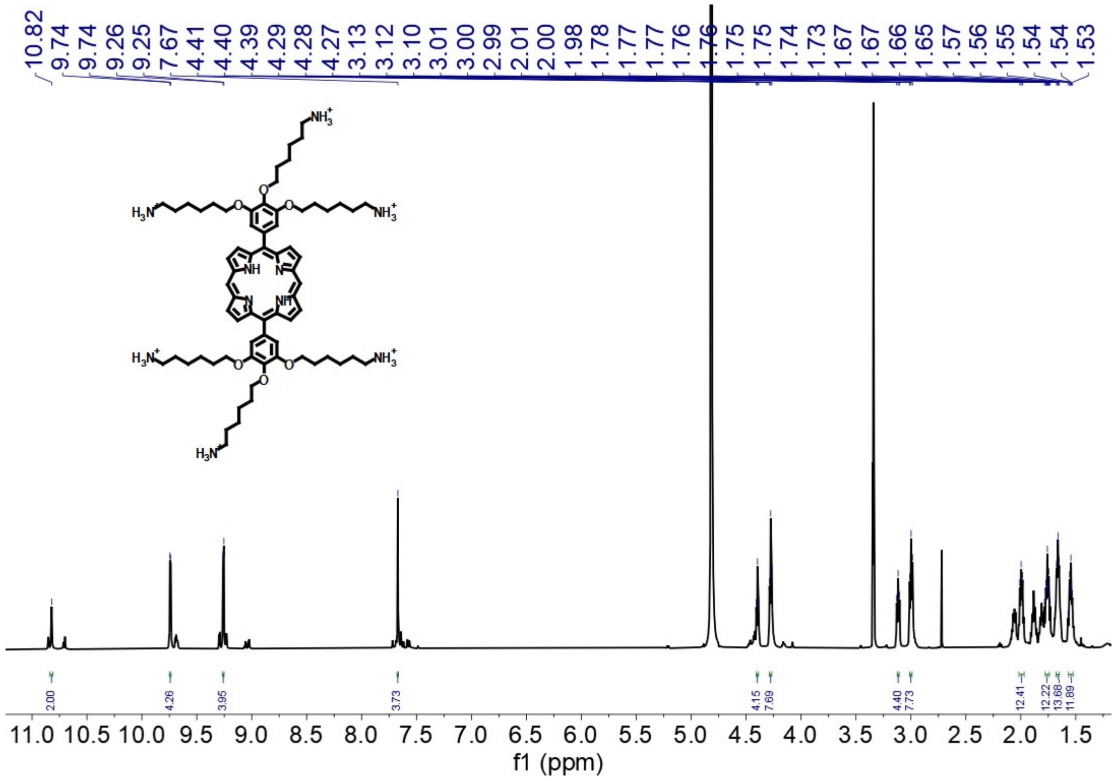

Figure S42. ^{1}H-NMR spectrum of **6C** in CD_3OD+D_2O.

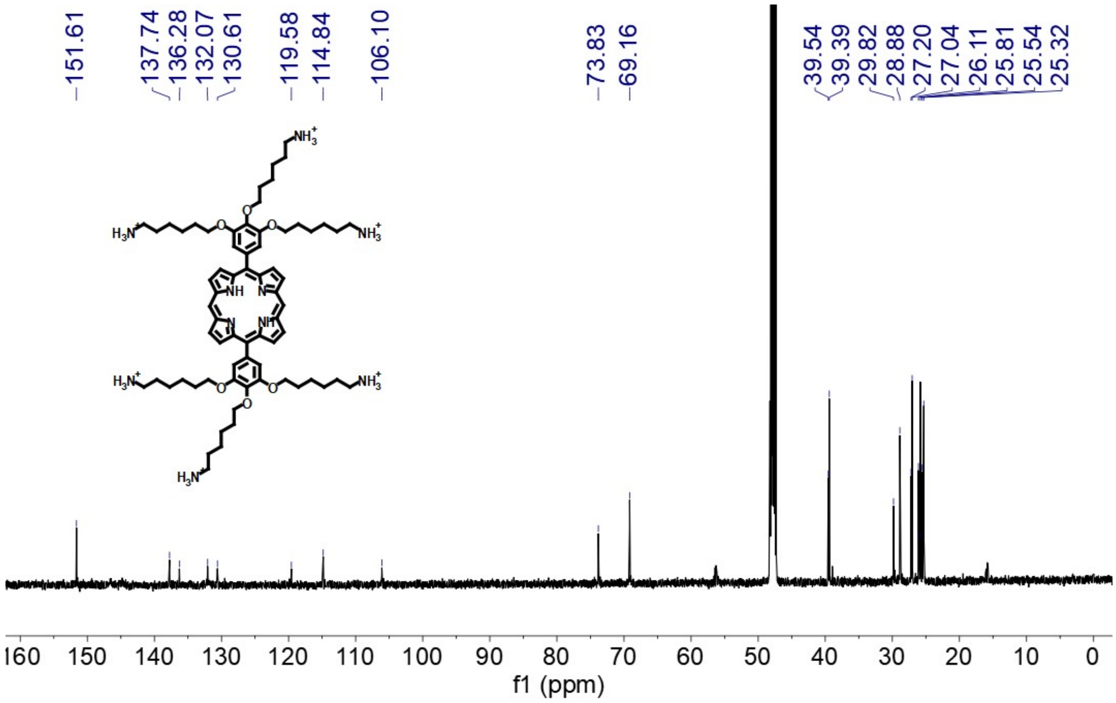

Figure S43. ^{13}C-NMR spectrum of **6C** in CD_3OD+D_2O.

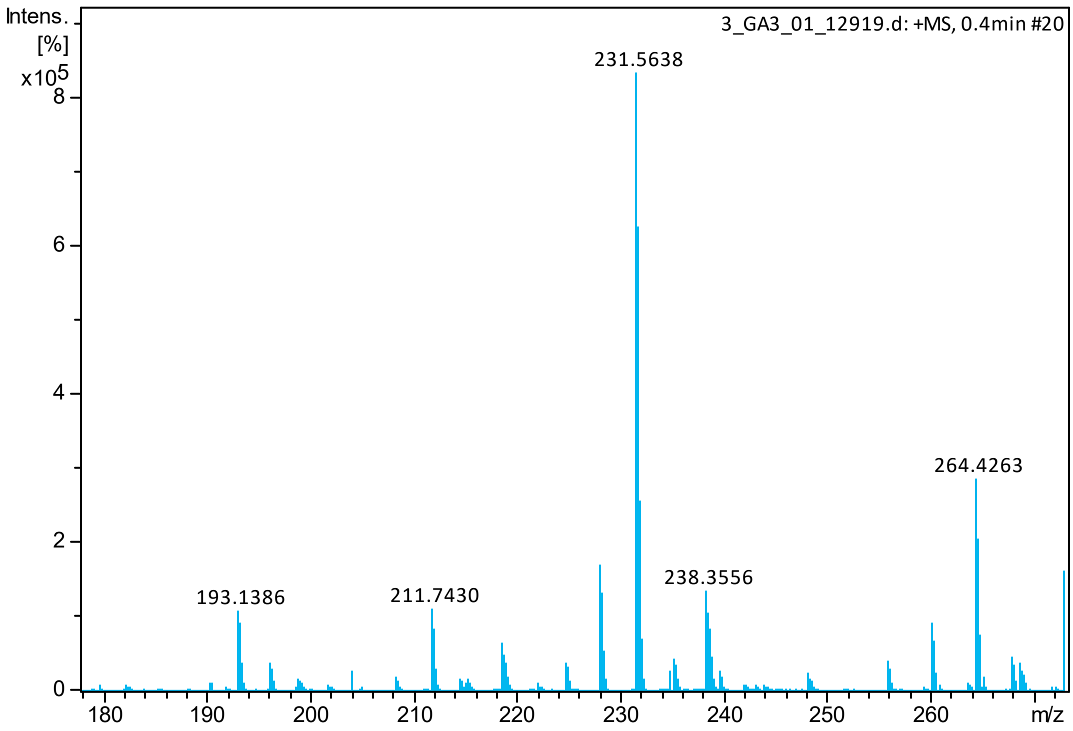

Figure S44. HRMS spectrum of **6C.**

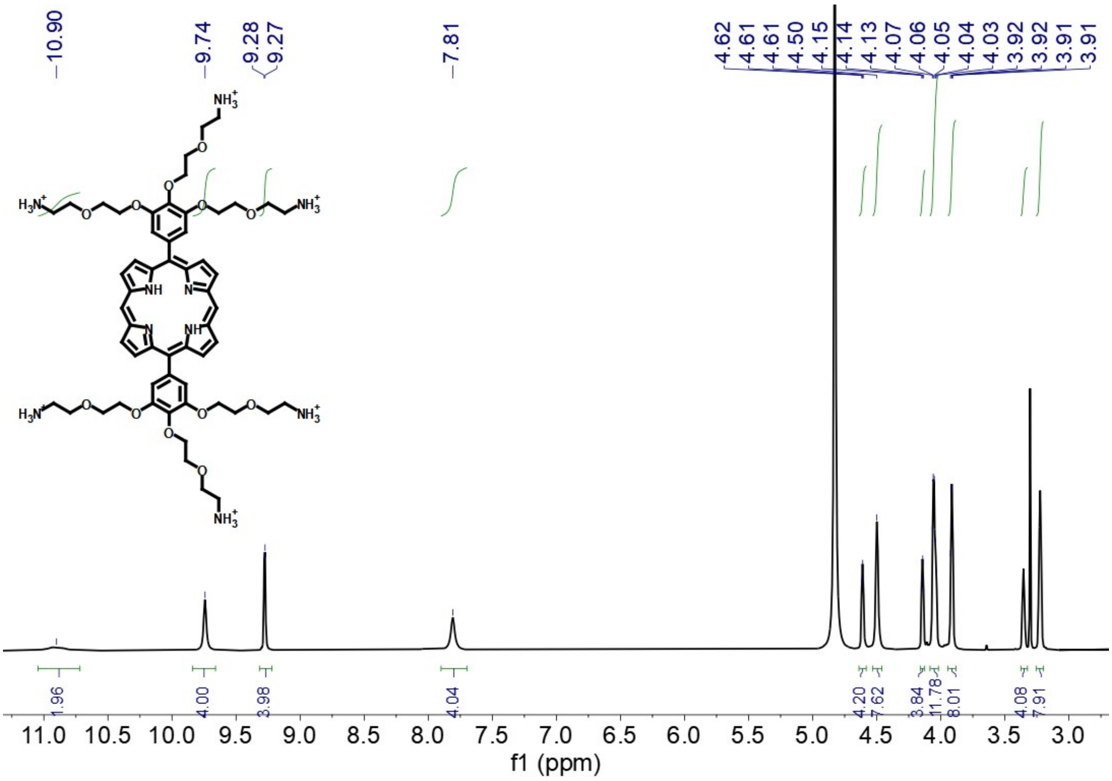

Figure S45. ^{1}H-NMR spectrum of **5C-O** in CD_3OD+D_2O.

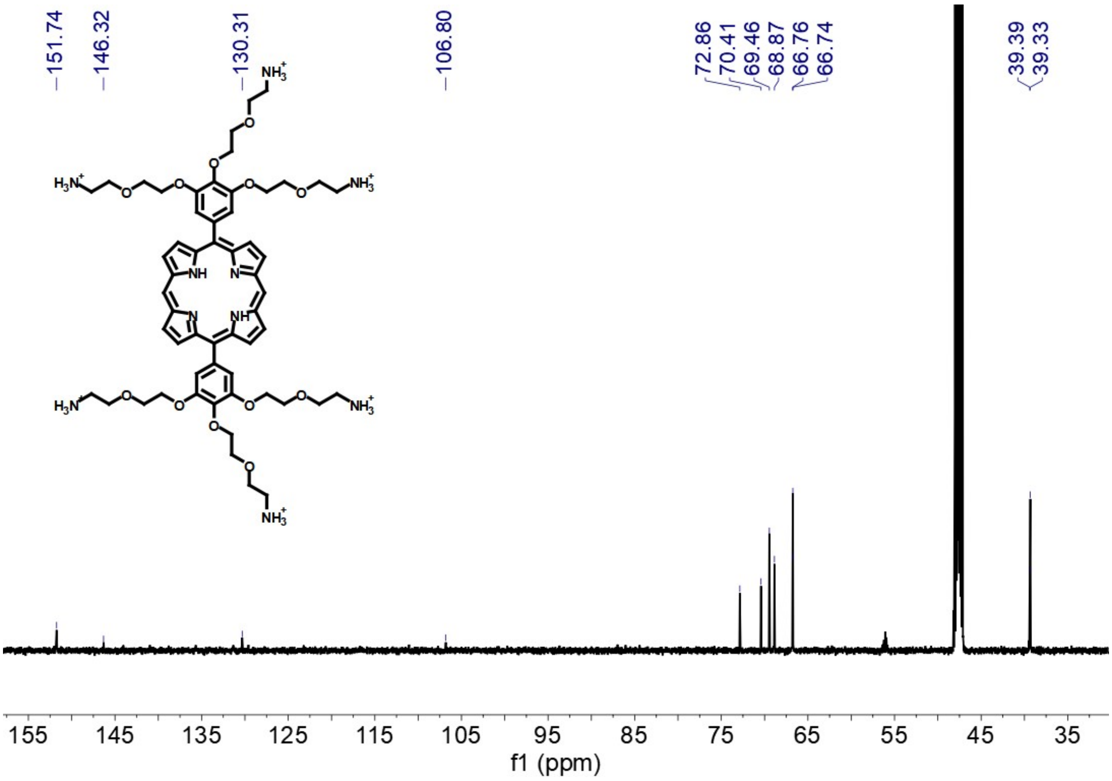

Figure S46. ^{13}C-NMR spectrum of **5C-O** in CD_3OD.

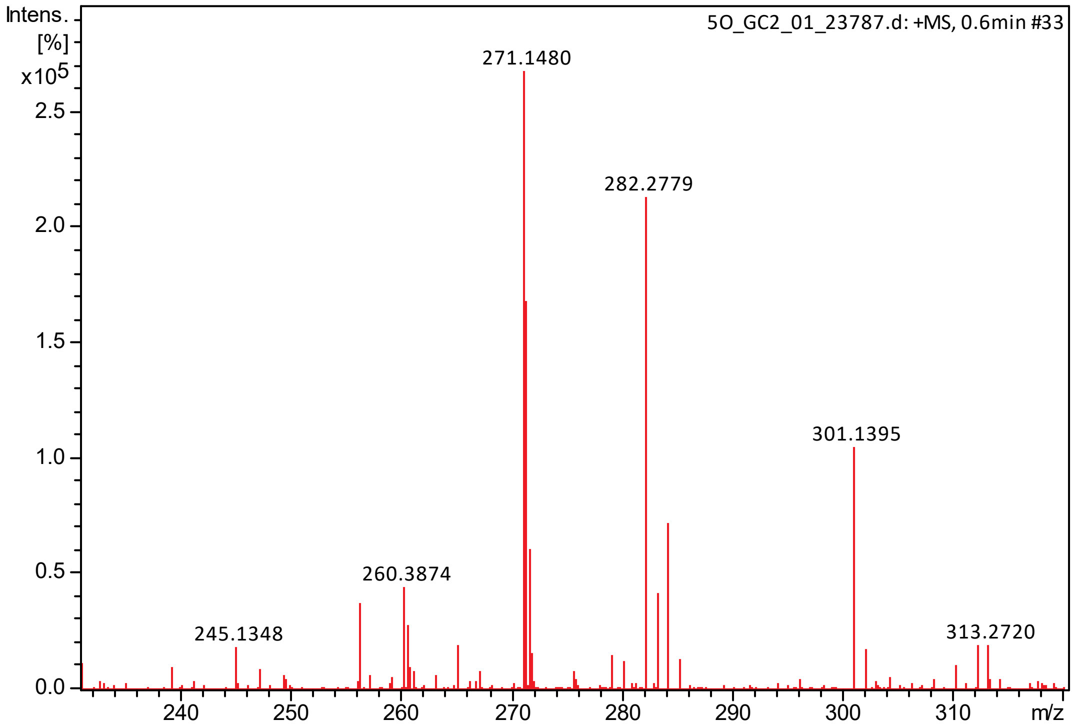

Figure S47. HRMS spectrum of **5C-O**.

6. References

1. Neese, F., Software Update: the ORCA Program System, Version 4.0. *WIREs Comput. Mol. Sci.* **2017**, *8*, e1327.

2. Grimme, S.; Ehrlich, S.; Goerigk, L., Effect of the Damping Function in Dispersion Corrected Density Functional Theory. *J. Comput. Chem.* **2011**, *32*, 1456-1465.

3. Stephens, P. J.; Devlin, F. J.; Chabalowski, C. F.; Frisch, M. J., Ab Initio Calculation of Vibrational Absorption and Circular Dichroism Spectra Using Density Functional Force Fields. *J. Phys. Chem.* **1994**, *98*, 11623-11627.

4. Izsák, R.; Neese, F., An Overlap Fitted Chain of Spheres Exchange Method. *J. Chem. Phys.* **2011**, *135*, 144105.

5. Neese, F.; Wennmohs, F.; Hansen, A.; Becker, U., Efficient, Approximate and Parallel Hartree–Fock and Hybrid DFT Calculations. A 'Chain-of-Spheres' Algorithm for the Hartree–Fock Exchange. *Chem. Phys.* **2009**, *356*, 98-109.

6. Knizia, G., Intrinsic Atomic Orbitals: An Unbiased Bridge between Quantum Theory and Chemical Concepts. *J. Chem. Theory Comput.* **2013**, *9*, 4834-4843.

7. Marenich, A. V.; Cramer, C. J.; Truhlar, D. G., Universal Solvation Model Based on Solute Electron Density and on a Continuum Model of the Solvent Defined by the Bulk Dielectric Constant and Atomic Surface Tensions. *J. Phys. Chem. B* **2009**, *113*, 6378-6396.

8. Donnier-Maréchal, M.; Carato, P.; Larchanché, P.-E.; Ravez, S.; Boulahjar, R.; Barczyk, A.; Oxombre, B.; Vermersch, P.; Melnyk, P., Synthesis and Pharmacological

Evaluation of Benzamide Derivatives as Potent and Selective Sigma-1 Protein Ligands. *Eur. J. Med. Chem.* **2017**, *138*, 964-978.
9. Spaeth, A.; Graeler, A.; Maisch, T.; Plaetzer, K., CureCuma–cationic Curcuminoids with Improved Properties and Enhanced Antimicrobial Photodynamic Activity. *Eur. J. Med. Chem.* **2018**, *159*, 423-440.
